# Antimicrobial Resistance in Six Priority Bacteria in Italy: Trends and Regional Variation, 2015–2024

**DOI:** 10.3390/antibiotics15090819

**Published:** 2026-08-23

**Authors:** Luigi Russo, Sofia Mao, Tindara Scirocco, Michela Sabbatucci, Andrea Zovi, Antonio Vitiello, Maria Rosaria Gualano, Walter Ricciardi, Leonardo Villani

**Affiliations:** 1 Section of Hygiene, Department of Life Sciences and Public Health, Università Cattolica del Sacro Cuore, Largo Francesco Vito 1, 00168 Rome, Italy; luigi.russo12@icatt.it (L.R.); tindara.scirocco01@icatt.it (T.S.); walter.ricciardi@unicatt.it (W.R.); leonardo.villani@unicatt.it (L.V.); 2 Department of Health Prevention, Research and Emergencies, Ministry of Health, Viale Giorgio Ribotta 5, 00144 Rome, Italya.vitiello@sanita.it (A.V.); 3 Department of Human, Animal and Ecosystem Health (One Health), and International Relations, Ministry of Health, Viale Giorgio Ribotta 5, 00144 Rome, Italy; a.zovi@sanita.it; 4 Faculty of Medicine, UniCamillus, Saint Camillus International University of Health and Medical Sciences, Via di Sant’Alessandro 8, 00131 Rome, Italy; mariarosaria.gualano@unicamillus.org

**Keywords:** antimicrobial resistance (AMR), rates of resistance, trend analysis, Italy

## Abstract

Background: Antimicrobial resistance (AMR) remains a major public health threat in Italy, where high incidence and mortality persist. In this study we aimed to describe national and regional trends in AMR rates from 2015 to 2024. Methods: We conducted an ecological time-series analysis using publicly available data from the Italian AMR surveillance system (AR-ISS). We included six priority pathogens: methicillin-resistant *Staphylococcus aureus* (MRSA), vancomycin-resistant *Enterococcus faecium* (VRE-*faecium*), third-generation cephalosporin-resistant *Escherichia coli* (3GCREC), carbapenem-resistant *Klebsiella pneumoniae* (CRKP), *Pseudomonas aeruginosa* (CRPA), and *Acinetobacter* spp. (CRAS). We analyzed annual resistance rates at national and regional level. Temporal trends were estimated using linear regression models, with calendar year as the independent variable. Regression coefficients, 95% CIs, and *p*-values were used to assess direction and significance. Relative percentage changes were calculated for 2015–2024 and, when relevant, for 2023–2024. Results: Resistance declined over time for four of six pathogens, but increased markedly for VRE-*faecium*, from 11.1% to 34.9%. Significant downward trends were observed for MRSA (β = −0.91, 95% CI: −1.35; −0.48, *p* = 0.0013), 3GCREC (β = −0.53, 95% CI: −0.99; −0.06, *p* = 0.0304), CRKP (β = −0.94, 95% CI: −1.35; −0.53, *p* = 0.0008), and CRPA (β = −0.86, 95% CI: −1.44; −0.27, *p* = 0.0095); VRE-*faecium* showed a strong upward trend (β = +2.76, 95% CI: 2.58; 2.94, *p* < 0.0001). CRAS remained consistently high throughout the study period. Considerable regional heterogeneity was observed across pathogens and geographical areas. Conclusions: Despite progress for several pathogens, AMR remains a critical challenge in Italy. The persistent increase in VRE-*faecium* and the high burden of CRAS support the need for strengthened surveillance, stewardship, and region-specific infection prevention interventions.

## 1. Introduction

Antimicrobial resistance (AMR) was recognized by the World Health Organization (WHO) in 2019 as one of the leading threats to global health, with more than 4 million deaths associated with bacterial AMR, including 1.4 million directly attributable to resistant infections [1,2]. AMR also generates a substantial economic burden for health systems through prolonged hospitalization, additional diagnostic and therapeutic interventions, and higher overall resource use [3].

In the European Union/European Economic Area (EU/EEA), AMR was responsible for approximately 35,800 deaths in 2020, an increase compared with 2016, when about 30,700 deaths were reported [4]. Italy carried a particularly high burden [5], accounting for almost one third of all deaths (11,000 deaths) in the EU/EEA [4].

The large-scale use of antibiotics often inappropriately prescribed [6], together with social, cultural, behavioural, economic, and health system factors [7], have exerted substantial selective pressure on bacterial populations promoting the emergence and spread of resistant strains [8,9,10]. Several pathogen–resistance combinations are of particular concern because of their contribution to the burden of antimicrobial resistance, their association with severe invasive infections, and the progressive restriction of effective therapeutic options. In particular, methicillin-resistant *Staphylococcus aureus* (MRSA), vancomycin-resistant *Enterococcus faecium* (VRE-*faecium*), third-generation cephalosporin-resistant *Escherichia coli* (3GCREC), carbapenem-resistant *Klebsiella pneumoniae* (CRKP), carbapenem-resistant *Pseudomonas aeruginosa* (CRPA), and carbapenem-resistant *Acinetobacter* spp. (CRAS) represent priority pathogen–resistance combinations for surveillance and public health action. These combinations capture both Gram-positive and Gram-negative resistance threats and include organisms associated with substantial healthcare burden, limited treatment options, and potential for transmission in healthcare settings [11].

To address the growing threat of AMR, the WHO adopted the Global Action Plan on AMR in 2015 [12], encouraging Member States to develop National Action Plans (NAPs) based on a One Health approach. In Italy, this framework was translated into the first NAP (PNCAR 2017–2020) [13], later extended to 2021 during the COVID-19 pandemic, and renewed as NAP 2022–2025 [14]. The plan sets out coordinated actions to strengthen surveillance, improve infection prevention and control, promote prudent antibiotic use, and support diagnostics, training, communication, research, and intersectoral coordination across human, animal, and environmental health, following the One Health approach. The implementation is organized around three core pillars: integrated surveillance systems; prevention and control of healthcare-associated and zoonotic infections; and antimicrobial stewardship in both human and veterinary settings. Overall, the plan aims to reduce the burden of AMR by improving data systems, reinforcing prevention capacity, and fostering more responsible use of antimicrobials.

In this context, temporal trend analysis is critical to understanding how resistance patterns evolve, where progress is being made, and where gains remain fragile or absent. Beyond describing the burden at a single point in time, it allows assessment of the direction, magnitude, and heterogeneity of change across pathogens and regions, offering a more policy-relevant basis for action [15,16,17,18,19]. In Italy, national AMR data are routinely collected through the AMR surveillance system coordinated by the National Institute of Health (AR-ISS) and reported annually [20]. While these reports provide essential descriptive surveillance data, they do not offer a unified, formal longitudinal comparison of temporal changes across pathogen–resistance combinations and regions. Such an assessment is particularly relevant in Italy, where observed regional variation may reflect differences in surveillance capacity, antimicrobial stewardship implementation, and infection prevention infrastructure. Therefore, this study systematically consolidates AR-ISS data from 2015 to 2024 and applies a consistent analytical framework to six priority pathogen–resistance combinations. By quantifying the direction and magnitude of national and regional trends, as well as recent variations, the analysis complements routine surveillance reporting and helps identify persistent challenges, emerging patterns, and regional priorities for targeted public health action.

## 2. Results

### 2.1. Descriptive Analysis of AMR Rates in Italy

Resistance rates for pathogen varied in Italy (Table 1) during the study period. Highest values were observed in 2022 for CRAS (88.5%), in 2024 for VRE-*faecium* (34.9%), in 2019 for MRSA (34.3%), in 2016 for CRKP (33.8%) and for CRPA (23.3%), and in 2019 for 3GCREC (30.9%). On the other hand, the lowest values were observed in 2015 for VRE-*faecium* (11.1%), in 2019 for CRPA (13.7%), in 2024 for CRKP (24.0%), in 2022 for 3GCREC (24.2%), in 2024 for MRSA (25.7%), and in 2024 for CRAS (74.3%). The total number of isolates and resistant isolates underlying the reported resistance percentages are provided in Supplementary Table S1, both nationally and by region.

### 2.2. Trends in Italy from 2015 to 2024

**Figure 1:**
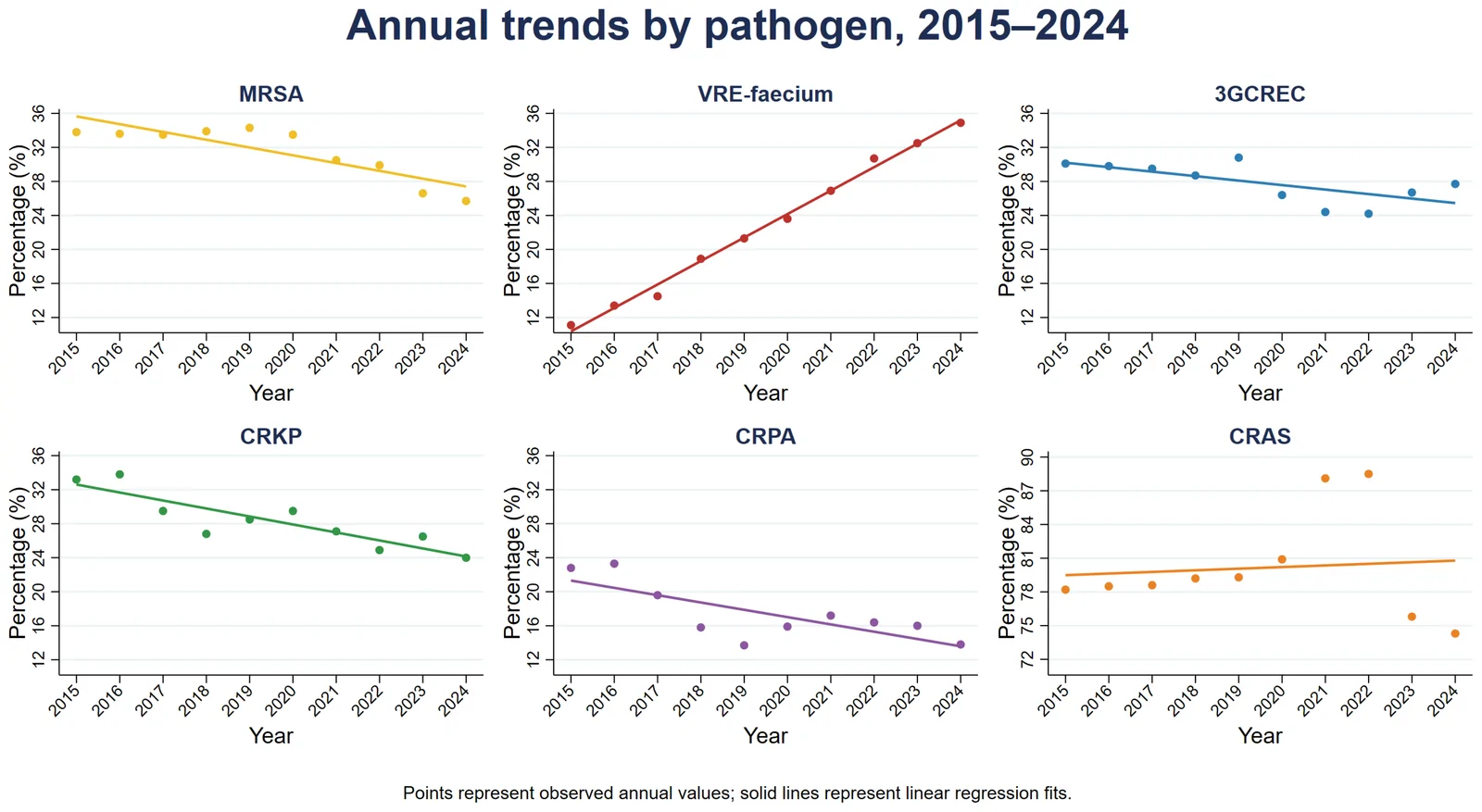
Linear regression models of antimicrobial-resistance in isolated species in Italy, from 2015 to 2024. MRSA = *Staphylococcus aureus* resistant to methicillin; VRE-*faecium* = *Enterococcus faecium* resistant to vancomycin; 3GCREC = *Escherichia coli* resistant to third-generation cephalosporins; CRKP = *Klebsiella pneumoniae* resistant to carbapenems; CRPA = *Pseudomonas aeruginosa* resistant to carbapenems; CRAS = *Acinetobacter species* resistant to carbapenems.

AMR trends in Italy between 2015 and 2024 varied notably across bacterial species (Figure 1). CRKP, MRSA, and CRPA demonstrated overall decreasing trends, whereas CRAS remained highly resistant for most of the period. In contrast, VRE-*faecium* continued to rise steadily, and 3GCREC showed moderate reductions with signs of plateauing. Significant decreases were observed for MRSA (β = −0.91, 95% CI: −1.35; −0.48, *p* = 0.0013), 3GCREC (β = −0.53, 95% CI: −0.99; −0.06, *p* = 0.0304), CRKP (β = −0.94, 95% CI: −1.35; −0.53, *p* = 0.0008) and CRPA (β = −0.86, 95% CI: −1.44; −0.27, *p* = 0.0095). CRAS showed a positive but not statistically significant increase (β = +0.14, 95% CI: −1.11; 1.40, *p* = 0.7977). Conversely, VRE-*faecium* showed a statistically significant annual increase (β = +2.76, 95% CI: 2.58; 2.94, *p* < 0.0001) (Table 2).

### 2.3. Trend of AMR Rates from 2015 to 2024, by Region

**Figure 2:**
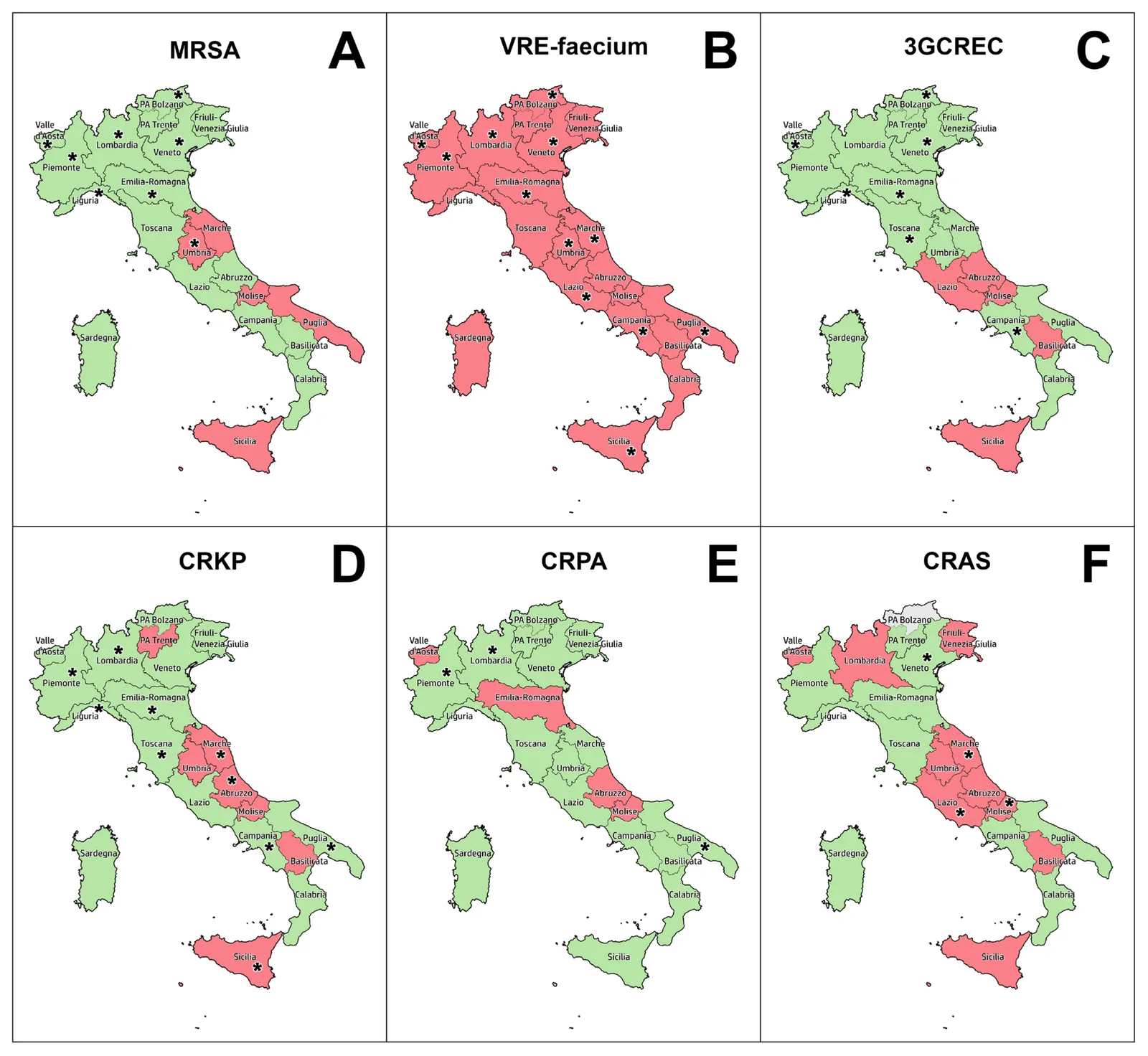
Trends in antimicrobial-resistance in isolated species in Italy, by pathogen identified by regression model, from 2015 to 2024. In red, increase; in green, decrease. * = significant trend. Grey = NA. MRSA = *Staphylococcus aureus* resistant to methicillin; VRE-*faecium* = *Enterococcus faecium* resistant to vancomycin; 3GCREC = *Escherichia coli* resistant to third-generation cephalosporins; CRKP = *Klebsiella pneumoniae* resistant to carbapenems; CRPA = *Pseudomonas aeruginosa* resistant to carbapenems; CRAS = *Acinetobacter species* resistant to carbapenems. (**A**): MRSA; (**B**): VRE-faecium; (**C**): 3GCREC; (**D**): CRKPL; (**E**): CRPA; (**F**): CRAS.

AMR trends across Italian regions revealed substantial variability depending on both pathogen and geography (Table 3). Table 2 summarizes the national regression estimates and the distribution of increasing and decreasing regional trends. Figure 2 provides a visual overview of the direction and statistical significance of regional temporal trends for each pathogen–resistance combination, whereas the complete regression estimates are reported in Supplementary Table S2.

For MRSA, an increasing trend was observed in five regions, while a decreasing trend was observed in 16 regions, seven of which were significant (Figure 2A). The greatest increases were observed in Umbria (β = 2.1, 95% CI: 0.55; 3.66, *p* = 0.0151), Sicilia (β = 0.85, 95% CI: −3.24; 4.94, *p* = 0.6446), and Molise (β = 0.69, 95% CI: −1.80; 3.19, *p* = 0.5213). The largest decreases were observed in Valle d’Aosta (β = −3.03, 95% CI: −4.46; −1.59, *p* = 0.0029), Veneto (β = −2.71, 95% CI: −3.89; −1.52, *p* = 0.0008), and Liguria (β = −2.5, 95% CI: −3.99; −1.02, *p* = 0.0045).

For VRE-*faecium*, increasing trends were observed in all regions, with 12 showing significant increases (Figure 2B). The steepest increases were found in Umbria (β = 6.4, 95% CI: 4.45; 8.35, *p* = 0.0001), Valle d’Aosta (β = 5.82, 95% CI: 2.32; 9.32, *p* = 0.0079), and Molise (β = 4.66, 95% CI: −3.07; 12.39, *p* = 0.1905). The lowest increase was observed in Friuli Venezia Giulia (β = 0.23, 95% CI: −2.18; 2.64, *p* = 0.8224).

For 3GCREC, trends declined in 16 regions, with seven showing significant decreases, and increased in five regions (Figure 2C). The greatest increases were found in Abruzzo (β = 0.72, 95% CI: −1.72; 3.16, *p* = 0.4819), Basilicata (β = 0.72, 95% CI: −0.87; 2.31, *p* = 0.3081), and Sicilia (β = 0.48, 95% CI: −0.98; 1.95, *p* = 0.4682). The most pronounced decreases were observed in Valle d’Aosta (β = −4.03, 95% CI: −6.86; −1.19, *p* = 0.0147), Liguria (β = −1.53, 95% CI: −2.38; −0.68, *p* = 0.0032), and Toscana (β = −1.42, 95% CI: −2.02; −0.82, *p* = 0.0006).

Regarding CRKP, seven regions showed increases, with three regions being significant, while the remaining 14 regions showed decreases, with seven regions being significant (Figure 2D). The highest increases were observed in Abruzzo (β = 6.19, 95% CI: 2.46; 9.92, *p* = 0.008), Sicilia (β = 5.74, 95% CI: 2.74; 8.73, *p* = 0.0022), and Basilicata (β = 2.64, 95% CI: −1.90; 7.17, *p* = 0.2048). Marked reductions were seen in Liguria (β = −4.79, 95% CI: −5.78; −3.80, *p* < 0.0001), Campania (β = −2.68, 95% CI: −3.91; −1.45, *p* = 0.001), and Toscana (β = −2.61, 95% CI: −3.49; −1.73, *p* = 0.0001).

For CRPA, four regions reported an increasing trend, none of which was significant, and 17 regions a decreasing trend (Figure 2E). The largest increases were observed in Abruzzo (β = 2.86, 95% CI: −0.04; 5.75, *p* = 0.0521), Valle d’Aosta (β = 1.19, 95% CI: −5.26; 7.65, *p* = 0.6347), and Molise (β = 0.69, 95% CI: −3.52; 4.90, *p* = 0.7010). The most notable reductions were observed in Liguria (β = −4.79, 95% CI: −5.78; 3.80, *p* < 0.0001), Calabria (β = −4.2, 95% CI: −11.23; 2.84, *p* = 0.1947), and Basilicata (β = −2.87, 95% CI: −8.40; 2.67, *p* = 0.2522).

Finally, for CRAS, increases were observed in 10 regions, three of which were significant, whereas 10 regions showed decreases, only one of which was significant (Figure 2F). The highest increases were found in Molise (β = 10.09, 95% CI: 3.44; 16.75, *p* = 0.0081), Abruzzo (β = 6.84, 95% CI: −10.59; 24.28, *p* = 0.3593), and Marche (β = 3.65, 95% CI: 0.49; 6.82, *p* = 0.0288). Marked reductions were observed in Trento (β = −6.06, 95% CI: −13.19; 1.07, *p* = 0.0855), Liguria (β = −2.81, 95%CI: −8.70; 3.08, *p* = 0.3037), and Veneto (β = −2.37, 95% CI: −4.73; 0.00, *p* = 0.0496). Visual representations of the regression analyses are provided in Supplementary Figures S1–S6.

## 3. Discussion

AMR trends in Italy emerging from our analysis reveal a complex epidemiological landscape characterized by both encouraging progress and persistent challenges. Overall, resistance rates declined over the study period for five of the six priority pathogens (CRKP, 3GCREC, CRPA, MRSA, and CRAS), although CRAS resistance remained alarmingly high (always ≥74%). In contrast, VRE-*faecium* exhibited a continuous increase, representing the most concerning finding of this analysis. Comparison between 2023 and 2024 also showed a decrease for all but 3GCREC and VRE-*faecium* that increased.

Despite these improvements, resistance rates remain substantially high and alarming when compared to European averages. According to ECDC data, in 2024 the mean CRKP resistance across Europe was 11.3%, roughly half of the Italian rate (24.0%) [21]. Similarly, the average European prevalence of VRE-*faecium* was 16.5%, compared with 34.9% in Italy [21]. McSorley et al. [22], analyzing EARS-Net data across 29 European countries from 2017 to 2021, found seven countries (Belgium, Denmark, Estonia, France, Ireland, Netherlands, and Norway) with no increase in MRSA, VRE-*faecium*, 3GCREC or CRKP, while Italy and Portugal had a high baseline resistance with reductions across most resistotypes, but a persistent rise in VRE-*faecium*. In Italy, this rise is likely driven by multiple interacting factors: Sacco et al. [23], analyzing 2015–2023 trends, found that large hospital sizes (>400 beds) and prolonged stays (>10 days) were independently associated with higher VRE-*faecium* odds; they also noted that the COVID-19 pandemic may have amplified spread of *Enterococcus* spp. infections in hospitalized patients, compounding the effect of broad-spectrum antibiotic pressure and existing stewardship gaps. Exposure to vancomycin, including oral vancomycin used for *Clostridioides difficile* infection, may also represent a potential selective pressure favouring VRE intestinal colonization [24,25]. However, our aggregate surveillance data do not include antibiotic-specific exposure or *Clostridioides difficile* infection data and therefore do not allow assessment of its contribution to the increasing VRE-*faecium* trend.

For CRPA, Tomic et al. [26] reported average resistance of 54.4% in Serbia and 56% in Romania during 2015–2020, compared with 4.4% in the Netherlands and 5.3% in Finland. In Italy, resistance declined from 22.8% to 15.9% over the same period, remaining below Serbia and Romania but above the Netherlands and Finland.

Regional differences highlight uneven AMR distribution nationwide, and may reflect variation in antimicrobial stewardship implementation, infection prevention and control practices, and antibiotic use [27]. Although some exceptions in northern regions (e.g., VRE-*faecium* in Piemonte (36.0%) and in Valle d’Aosta (42.1%); MRSA (27.8%) in Lombardia), southern and centre regions generally exhibited higher resistance rates for selected pathogens, both in long-term trends and in the most recent years. Regional differences may partly reflect variation in surveillance coverage. In 2024, 210 laboratories contributed to the AR-ISS network, corresponding to a national coverage of 67.5%. Coverage exceeded 75% in twelve regions, including only three southern regions (Basilicata, Sicilia, and Sardegna). Differences in laboratory participation, surveillance organization, laboratory capacity, and reporting practices may therefore contribute to the observed heterogeneity, alongside genuine epidemiological differences between regions [28].

Several factors may contribute to the persistently high resistance levels observed in Italy. The country has historically faced high antimicrobial consumption and challenges in antimicrobial stewardship, awareness, and governance, as highlighted by the ECDC country assessment in 2017 [29]. Even though modest decline has been observed, the recent increase in 3GCREC and VRE-*faecium* may signal a reversal of previous gains, also reflecting the residual impact of the COVID-19 pandemic on surveillance and stewardship activities [30,31]. This is also particularly relevant for the economic burden of AMR [32], since these infections require costly therapies such as linezolid, daptomycin [33], or complex combination regimens [34]. Nevertheless, the overall decline observed for most pathogens provides encouraging evidence of progress and suggests that the strengthening of national AMR policies and coordinated interventions may be contributing to improved control.

To promote a more coordinated effort, Italy joined the European Joint Action on Antimicrobial Resistance and Healthcare-Associated Infections (EU-JAMRAI) [35], and in 2022, the Ministry of Health issued the updated National Action Plan on Antimicrobial Resistance (PNCAR 2022-25) [36] to strengthen surveillance and stewardship activities.

Moving forward, Italy must strengthen its AMR monitoring systems through mandatory national reporting, standardized data flows, and accountability metrics to ensure implementation across regions [37,38,39]. The establishment of early warning systems (EWSs) for emerging resistance profiles could allow prompt detection and containment of novel or high-risk AMR patterns [38]. Persistent regional disparities call for tailored interventions that reflect local epidemiological and organizational contexts [40].

Specific control measures targeting VRE-*faecium* and CRAS should prioritize strengthened environmental hygiene protocols in healthcare facilities, active screening of high-risk patients, and optimized antibiotic formulary restrictions, supported by infection prevention teams [40,41,42]. At the system level, implementing control frameworks for stewardship and IPC activities across hospitals and regional authorities is essential to translate national objectives into measurable local actions [37,43].

Reducing inappropriate antibiotic prescribing remains a central challenge. Recent analyses [44] indicate persistently high outpatient antibiotic consumption, driven by inappropriate use in primary care, particularly in southern regions [45]. In this context, the PNCAR represents an important policy instrument, having contributed to reducing antibiotic prescribing in primary care, although its impact is likely to depend on sustained implementation and integration with complementary interventions to effectively tackle AMR [46].

For example, education programmes, targeting both healthcare professionals and patients, and public awareness campaigns can help reduce unnecessary prescriptions and increase adherence to evidence-based guidelines [47,48].

This study should be interpreted considering certain limitations. First, regional participation in the AR-ISS surveillance remains heterogeneous, potentially affecting national representativeness and bias estimates toward regions with more robust surveillance capacity. Differences in AMR awareness may influence surveillance intensity and diagnostic practices, as well as testing practices, with some microorganisms being tested less frequently than others, and testing may be preferentially performed in clinical contexts associated with a higher suspicion of AMR. This appears to be the case for CRAS. Moreover, differences in laboratory methodologies, such as testing panels, may also introduce variability. Second, substantial regional variations in data availability, particularly in earlier years of detection, limit the precision of longitudinal trend analyses and may reduce the ability to fully capture non-linear changes over time. Our study period also encompasses the COVID-19 pandemic, which likely influenced both antimicrobial resistance patterns and surveillance activities. Third, the absence of patient-level clinical outcomes prevents direct assessment of the impact of AMR trends on individual outcomes, limiting causal interpretation. Finally, this study focuses on the Italian context and its regional organization, and it is not directly generalizable to other EU countries and settings. However, it is important to recognize that Italy is the third most populous country in EU, and its territory encompasses large and highly heterogeneous populations. Some Italian regions (e.g., Lombardia) are comparable to entire EU countries (e.g., Belgium or Portugal) in terms of population size and economic output, underscoring that they represent territories with substantial demographic and organizational complexity, which need to be analyzed and addressed individually.

Despite these limitations, this study provides a comprehensive and policy-relevant overview of AMR trends in Italy over the last decade, highlighting persistent critical priorities and offering robust evidence to inform national surveillance strengthening, antimicrobial stewardship, and infection prevention strategies in one of the European countries most heavily burdened by AMR.

## 4. Materials and Methods

We conducted an observational ecological time-series analysis based on nationally aggregated data, reported in accordance with the STROBE guidelines (Supplementary File S1) [49].

### 4.1. The Italian AMR Surveillance System (AR-ISS)

Data on resistance rates were extracted from the AR-ISS (antimicrobial resistance) surveillance system) by the National Institute of Health, specifically from the annual reports “AR-ISS: Sorveglianza Nazionale dell’Antibiotico-Resistenza” [20,28]. The AR-ISS system collects data through a national network of hospital-based clinical microbiology laboratories coordinated by the Italian National Institute of Health (ISS). Participating laboratories, recruited through regional health authorities, routinely perform antimicrobial susceptibility testing (AST) as part of their diagnostic activities and annually transmit results for selected bacterial species isolated from invasive infections. Participation is voluntary but supported by regional surveillance networks, ensuring progressively wider geographical representativeness. The central coordination unit at ISS validates, harmonizes, and analyses the data, applying standardized interpretive criteria before aggregating national and regional resistance trends. Data from AR-ISS constitute the Italian contribution to the European Antimicrobial Resistance Surveillance Network (EARS-Net) and, since 2020, to the WHO Global Antimicrobial Resistance and Use Surveillance System (GLASS).

### 4.2. Data Collection

We considered the period from 2015 (or the first available year for each region) to 2024.

We included AMR data presented as the percentage of resistant isolates relative to the total number of isolates identified. We collected data for the six priority organisms and their relative resistance rates: Methicillin-Resistant *Staphylococcus aureus* (MRSA), Vancomycin-Resistant *Enterococcus faecium* (VRE-*faecium*), third-generation Cephalosporin-Resistant *Escherichia coli* (3GCREC), Carbapenem-Resistant *Klebsiella pneumoniae* (CRKP), Carbapenem-Resistant *Pseudomonas aeruginosa* (CRPA), and Carbapenem-Resistant *Acinetobacter species* (CRAS) are recognized among the most critical bacteria.

The AR-ISS protocol specifies a minimum panel of antibiotics to be tested for each bacterial species, focusing on antibiotics of therapeutic or epidemiological relevance [50].

Resistance categories were extracted from the annual AR-ISS reports and were based on routine phenotypic antimicrobial susceptibility testing; molecular resistance markers were not used in the present analysis. According to the most recent AR-ISS reporting definitions [51], MRSA was defined as *Staphylococcus aureus* resistant to at least one of oxacillin or cefoxitin; VRE-*faecium* as *Enterococcus faecium* resistant to vancomycin; 3GCREC as *Escherichia coli* resistant to at least one of cefotaxime, ceftazidime, or ceftriaxone; and CRKP, CRPA, and CRAS as *Klebsiella pneumoniae*, *Pseudomonas aeruginosa*, and *Acinetobacter* spp., respectively, resistant to at least one of imipenem or meropenem.

The AR-ISS and EARS-Net protocols, including the antimicrobial testing panels used to define some resistance categories, were updated during the study period. Therefore, the analyses retained the operational resistance categories reported by AR-ISS for each year, without retrospectively reclassifying isolates according to a single harmonized microbiological definition across the entire study period.

Data were collected at both national and regional levels. For the regional analyses, we used the resistance data presented by region in the annual AR-ISS reports. These regional aggregates are generated from data submitted by participating hospital microbiology laboratories and, in some regions, by regional AMR surveillance networks. Therefore, regional estimates should be interpreted as surveillance-based indicators for the relevant region, rather than as patient residence-based estimates. Differences in laboratory participation, surveillance coverage, and regional data-reporting arrangements should be considered when interpreting regional heterogeneity.

According to the AR-ISS protocol [50], when repeated isolates of the same pathogen from the same patient and specimen type are identified during the same calendar year, only the first isolate is included in the surveillance dataset, while subsequent duplicates are excluded.

### 4.3. Data Analysis

Descriptive analyses of AMR trends were performed at both national and regional levels, evaluating percentage changes over the study period. Specifically, we examined changes in resistance rates by calculating the relative percentage variation as follows:

$$\Delta =\;\frac{\left(Resistance\;rate\;of\;the\;later\;year-\;Resistance\;rate\;of\;the\;earlier\;year\right)}{Resistance\;rate\;of\;the\;earlier\;year}\times 100$$

Variations were calculated to assess differences between the first available year and the most recent one (2015 to 2024), as well as changes over the last two years (2023 and 2024). For regions where data are available starting from years after 2015, variations were calculated starting from the first available year, as specified. When the first available year showed no resistant pathogens isolated, the variation for that region–pathogen was not calculable.

To identify regions with higher or lower resistance levels, we described resistance percentages in 2024 for each microorganism at regional level. At national level, data were analyzed as temporal trends to assess year-to-year fluctuations, while regional trends were evaluated to facilitate inter-regional comparisons. Trend analyses were performed using separate linear regressions of resistance rates on calendar year for each Italian region, estimating annual trends and assessing whether resistance rates significantly changed over the 2015–2024 period for each organism.

The distribution of variables was assessed using the Shapiro–Wilk test for normality of the residuals. Visual inspection was also performed using Q–Q plots and histograms with overlaid normal curves to evaluate the assumption of normality and identify potential deviations from a normal distribution.

All statistical analyses were performed using Stata version 17 (StataCorp, College Station, TX, USA). As the analyses were conducted using standard Stata procedures, no additional packages were required. The analysis code is available from the corresponding authors upon reasonable request.

## 5. Conclusions

AMR in Italy remains a major public health challenge, with persistently high CRAS resistance and a concerning increase in VRE-*faecium*. Nevertheless, the decline observed for all priority pathogens other than VRE-*faecium* represents an encouraging signal of progress, suggesting that the measures implemented in recent years are having a positive impact, although further action is still needed.

By systematically synthesizing AR-ISS, this study provides an additional and more accessible perspective to the annual surveillance reports, supporting evidence-based decision-making. Future efforts should include regular updating of these analyses and continued improvement of the AR-ISS system, particularly in terms of regional coverage. A stronger surveillance system would also facilitate the identification of effective regional prevention and stewardship strategies and the transfer of best practices across regions. Together, these actions are essential to consolidate the progress achieved and address the remaining and emerging AMR threats in Italy.

## Figures and Tables

**Table 1:** Rates of antimicrobial-resistance in isolated species in Italy, from 2015 to 2024.

Pathogen–Resistance Combination	Metric	2015	2016	2017	2018	2019	2020	2021	2022	2023	2024	Δ (%) 2015–2024	Δ (%) 2023–2024
MRSA	I (N.)	3037	3018	3634	8323	9682	10,976	12,191	14,083	14,943	16,226		
	R (N.)	1027	1014	1219	2823	3322	3673	3716	4206	3969	4163		
	**R (%)**	**33.8**	**33.6**	**33.5**	**33.9**	**34.3**	**33.5**	**30.5**	**29.9**	**26.6**	**25.7**	**−23.96**	**−3.38**
VRE-*faecium*	I (N.)	767	944	1063	2291	2839	4177	5202	5910	6022	6323		
	R (N.)	85	126	154	434	605	984	1399	1815	1958	2206		
	**R (%)**	**11.1**	**13.3**	**14.5**	**18.9**	**21.3**	**23.6**	**26.9**	**30.7**	**32.5**	**34.9**	**+214.41**	**+7.08**
3GCREC	I (N.)	5593	5941	7079	16,253	18,410	18,760	21,920	25,683	28,581	31,649		
	R (N.)	1682	1770	2089	4664	5682	4955	5341	6223	7625	8775		
	**R (%)**	**30.1**	**29.8**	**29.5**	**28.7**	**30.9**	**26.4**	**24.4**	**24.2**	**26.7**	**27.7**	**−7.97**	**+3.75**
CRKP	I (N.)	1999	2304	2633	5661	7325	8282	9297	11,248	13,664	15,331		
	R (N.)	664	779	778	1516	2086	2447	2524	2801	3615	3673		
	**R (%)**	**33.2**	**33.8**	**29.5**	**26.8**	**28.5**	**29.5**	**27.1**	**24.9**	**26.5**	**24.0**	**−27.71**	**−9.43**
CRPA	I (N.)	1082	1206	1433	3014	3794	4616	5047	5972	6645	7374		
	R (N.)	247	281	281	476	521	736	866	981	1065	1014		
	**R (%)**	**22.8**	**23.3**	**19.6**	**15.8**	**13.7**	**15.9**	**17.2**	**16.4**	**16** **.** **0**	**13.8**	**−39.47**	**−13.75**
CRAS	I (N.)	665	706	869	1383	1588	2550	3297	2744	3103	3319		
	R (N.)	520	554	683	1096	1259	2064	2905	2429	2353	2465		
	**R (%)**	**78.2**	**78.5**	**78.6**	**79.2**	**79.3**	**80.9**	**88.1**	**88.5**	**75.8**	**74.3**	**−4.99**	**−1.98**

I (N.) = number of isolates; R (N.) = number of resistant isolates; R (%) = percentage of resistance. Resistance rates are expressed as percentages. The corresponding numbers of resistant isolates and total isolates are reported as I (N)/R (N). Δ represents the relative percentage change between the corresponding years, calculated as [(resistance rate in the later year − resistance rate in the earlier year)/resistance rate in the earlier year] × 100. MRSA = *Staphylococcus aureus* resistant to methicillin; VRE-*faecium* = *Enterococcus faecium* resistant to vancomycin; 3GCREC = *Escherichia coli* resistant to third-generation cephalosporins; CRKP = *Klebsiella pneumoniae* resistant to carbapenems; CRPA = *Pseudomonas aeruginosa* resistant to carbapenems; CRAS = *Acinetobacter species* resistant to carbapenems.

**Table 2:** National resistance trends and regional distribution of increasing and decreasing trends for six priority pathogen–resistance combinations in Italy, 2015–2024.

Pathogen–Resistance Combination	National Trend			Regional Trend Distribution					
	β Coefficent	95% CI	*p*-Value	Significant Increase	Non-Significant Increase	Increasing Trend	Significant Decrease	Non-Significant Decrease	Decreasing Trend
MRSA	−0.91	−1.35 to −0.48	0.0013	1	4	5	7	9	16
VRE-*faecium*	2.76	2.58 to 2.94	<0.0001	12	9	21	0	0	0
3GCREC	−0.53	−0.99 to −0.06	0.0304	0	5	5	7	9	16
CRKP	−0.94	−1.35 to −0.53	0.0008	3	4	7	7	7	14
CRPA	−0.86	−1.44 to −0.27	0.0095	0	4	4	3	14	17
CRAS	0.14	−1.11 to 1.40	0.7977	3	7	10	1	9	10

Note: CRAS data were not available for Bolzano.

**Table 3:** Differences in rates of antimicrobial-resistance in isolated species in Italy, between 2015 and 2024, and between the last two years available (2023–2024), by regions.

Regions	MRSA		VRE-*faecium*		3GCREC		CRKP		CRPA		CRAS	
	Δ (%) 2015-24	Δ (%) 2023-24	Δ (%) 2015-24	Δ (%) 2023-24	Δ (%) 2015-24	Δ (%) 2023-24	Δ (%) 2015-24	Δ (%) 2023-24	Δ (%) 2015-24	Δ (%) 2023-24	Δ (%) 2015-24	Δ (%) 2023-24
Abruzzo	−9.2 ^c^	+30.3	+26.3 ^c^	+12.9	+13.4 ^c^	+8.8	+159.4 ^c^	+2.7	+128.4 ^c^	−42.9	−2.0 ^c^	−6.6
Basilicata	−15.9 ^b^	−17.9	N.C.	+25.3	+16.5 ^b^	+13.6	+152.7 ^b^	+35.2	−6.2 ^b^	+35.0	+46.6 ^b^	−19.4
Calabria	−26.7 ^b^	+16.7	+70.6 ^b^	+10.1	+11.6 ^b^	+11.9	−33.9 ^b^	−10.3	−63.7 ^b^	+37.0	−9.5 ^b^	−1.0
Campania	−11.3	+1.6	+1707.1	−0.4	−14.6	+4.7	−43.0	−10.7	−52.5	−33.9	−2.5	+0.2
Emilia-Romagna	−37.1	−13.8	+1164.3	+2.0	−21.9	+10.0	−46.7	+10.8	−29.4	−35.9	−38.0	−28.4
Friuli Venezia Giulia	−12.7 ^b^	+9.3	+90.8 ^b^	+9.0	−4.6 ^b^	+18.6	−44.0 ^b^	−12.5	−6.5 ^b^	+33.3	N.C.	−70.0
Lazio	−16.2	−6.6	+143.1	−0.8	+13.6	+1.4	−23.6	−4.5	−18.6	−24.9	+27.0	−1.9
Liguria	−51.7	−6.0	+55.9	−33.4	−31.2	+10.2	−77.4	+10.8	−30.0	+8.5	−40.0	−32.1
Lombardia	−14.7	+3.3	+97.4	+10.5	−5.7	−2.1	−45.8	−17.9	−44.8	−17.2	+8.9	+15.6
Marche	+6.7	+22.8	N.C.	+5.7	+4.7	+11.2	+12.1	+7.2	−44.2	−20.2	+21.1	−2.2
Molise	+38.4 ^b^	+5.4	N.C.	+23.6	+6.6 ^b^	−3.0	+98.7 ^b^	+50.0	+24.6 ^b^	+52.9	+21.1 ^b^	+2.3
Bolzano	−49.5	−41.0	+331.0	+83.2	−29.3	+6.9	N.C.	−20.0	+21.0	+92.2	N.C.	N.C.
Trento	−66.5	−27.6	+123.1	+27.6	−11.9	−4.8	−23.1	−71.4	−47.3	−6.4	−100.0	N.C.
Piemonte	−41.6	+3.6	+44.0	−14.5	+4.3	−2.2	−44.6	−33.9	−50.0	+10.3	−47.1	−31.7
Puglia	+76.2	−0.4	N.C.	0.0	−4.4	+2.2	−30.7	−3.1	−47.4	−11.6	−17.3	−20.2
Sardegna	−8.7	−9.2	+62.7	−7.7	−12.8	+24.2	−4.2	−17.9	−35.4	−21.2	−17.1	−24.7
Sicilia	+33.6	−15.7	N.C.	+39.4	−4.8	+1.0	+386.1	−4.3	−56.3	+10.8	−5.0	+3.9
Toscana	−5.4	+9.0	+83.6	+23.0	−26.3	−2.5	−48.6	−3.2	−52.4	−12.8	−15.0	−0.5
Umbria	+49.4 ^a^	−0.8	+210.2 ^a^	+3.1	+0.39 ^a^	+0.8	+1.96 ^a^	−19.3	−30.50 ^a^	+6.5	+20.45 ^a^	−0.5
Valle d’Aosta	−35.2 ^c^	+21.1 ^c^	+62.0 ^c^	+38.5	−25.3 ^c^	−31.1	+18.6 ^c^	+3.6	N.C.	N.C.	+16.5 ^c^	N.C.
Veneto	−43.6	−9.4	+412.5	+26.2	−7.0	+9.0	−54.0	−35.4	+39.1	−6.2	−37.8	−7.3

Δ represents the relative percentage change between the corresponding years, calculated as [(resistance rate in the later year—resistance rate in the earlier year)/resistance rate in the earlier year] × 100. First available year was 2015 unless otherwise specified. ^a^ = from 2016; ^b^ = from 2017; ^c^ = from 2018. N.C. = not calculable since the first year considered has a real value of zero resistant pathogen isolated. MRSA = *Staphylococcus aureus* resistant to methicillin; VRE-*faecium* = *Enterococcus faecium* resistant to vancomycin; 3GCREC = *Escherichia coli* resistant to third-generation cephalosporins; CRKP = *Klebsiella pneumoniae* resistant to carbapenems; CRPA = *Pseudomonas aeruginosa* resistant to carbapenems; CRAS = *Acinetobacter species* resistant to carbapenems.

## Data Availability

The data used in this study are publicly available from the Italian National Institute of Health (ISS) through the AR-ISS National Antibiotic-Resistance Surveillance system. Specifically, the report “AR-ISS: National Antibiotic-Resistance Surveillance. Data 2023”, was used as the primary data source and is accessible at https://www.epicentro.iss.it/antibiotico-resistenza/ar-iss/RIS-5_2024.pdf (accessed on 17 October 2025). Data from 2024 are available at https://www.epicentro.iss.it/antibiotico-resistenza/ar-iss-rapporto (accessed on 17 October 2025). Moreover, all the data used for the analysis are available in Supplementary Table S1.

## Supplementary Materials

The following supporting information can be downloaded at https://www.mdpi.com/article/10.3390/antibiotics15090819/s1, Table S1: Number of isolates, resistant isolates, and resistance percentages for the analysed pathogen–antibiotic combinations in Italy, by year and by region; Table S2: Linear regression models of antimicrobial resistance in isolated species in Italy, by region and species, from 2015 to 2024; Figure S1: Linear regression models of MRSA resistance in Italy, by region; Figure S2: Linear regression models of VRE-*faecium* resistance in Italy, by region; Figure S3: Linear regression models of 3GCREC resistance in Italy, by region; Figure S4: Linear regression models of CRKP resistance in Italy, by region; Figure S5: Linear regression models of CRPA resistance in Italy, by region; Figure S6: Linear regression models of CRAS resistance in Italy, by region; File S1: STROBE Statement–Checklist of items that should be included in reports of observational studies.
