# Supplementary material for: Antimicrobial Resistance in Six Priority Bacteria in Italy: Trends and Regional Variation, 2015–2024
Source: Antibiotics (Basel). 2026 Aug 23;15(9):819. doi: 10.3390/antibiotics15090819 (PMC13603502; doi:10.3390/antibiotics15090819)
Supplement: Supplementary file 1 [file antibiotics-15-00819-s001.zip › antibiotics-4469150-supplementary.pdf]

Supplementary Table S1. Number of isolates, resistant isolates, and resistance percentages for the analysed pathogen–antibiotic combinations in Italy, by year and by region.

Region	Species	Metric	2015	2016	2017	2018	2019	2020	2021	2022	2023	2024
**Abruzzo**	**MRSA**	I (N.)	0	0	0	132	156	139	10	138	140	108
		R (N.)	0	0	0	50	39	59	8	49	37	37
		R (%)	.	.	.	37.9	25.0	42.4	80	35.5	26.4	34.3
	**VRE-*faecium***	I (N.)	0	0	0	48	59	75	22	130	122	108
		R (N.)	0	0	0	19	26	15	5	34	54	54
		R (%)	.	.	.	39.6	44.1	20.0	22.7	26.2	44.3	50.0
	**3GCREC**	I (N.)	0	0	0	206	209	159	282	354	426	412
		R (N.)	0	0	0	63	55	52	57	104	136	143
		R (%)	.	.	.	30.6	26.3	32.7	20.2	29.4	31.9	34.7
	**CRKP**	I (N.)	0	0	0	75	0	83	18	63	146	176
		R (N.)	0	0	0	12	0	17	5	23	59	73
		R (%)	.	.	.	16.0	.	20.5	27.8	36.5	40.4	41.5
	**CRPA**	I (N.)	0	0	0	45	0	57	69	93	112	150
		R (N.)	0	0	0	3	0	7	10	12	30	23
		R (%)	.	.	.	6.7	.	12.3	14.5	12.9	26.8	15.3
	**CRAS**	I (N.)	0	0	0	56	0	85	112	116	154	134
		R (N.)	0	0	0	52	0	80	109	112	150	122
		R (%)	.	.	.	92.9	.	94.1	97.3	96.6	97.4	91.0
**Basilicata**	**MRSA**	I (N.)	0	0	45	42	108	90	99	115	126	132
		R (N.)	0	0	15	13	37	34	40	41	43	37
		R (%)	.	.	33.3	31.0	34.3	37.8	40.4	35.7	34.1	28.0
	**VRE-*faecium***	I (N.)	0	0	5	8	26	31	38	45	40	47
		R (N.)	0	0	0	2	6	11	11	10	9	17
		R (%)	.	.	.	25.0	23.1	35.5	28.9	22.2	22.5	36.2
	**3GCREC**	I (N.)	0	0	67	71	122	107	111	122	150	211
		R (N.)	0	0	24	21	46	40	32	43	55	88
		R (%)	.	.	35.8	29.6	37.7	37.4	28.8	35.2	36.7	41.7
	**CRKP**	I (N.)	0	0	18	24	49	60	56	72	94	123
		R (N.)	0	0	4	12	21	24	37	31	39	69

		R (%)	.	.	22.2	50.0	42.9	40.0	66.1	43.1	41.5	56.1
	**CRPA**	I (N.)	0	0	13	14	22	12	17	29	28	38
		R (N.)	0	0	4	7	8	2	0	4	6	11
		R (%)	.	.	30.8	50.0	36.4	16.7	.	13.8	21.4	28.9
	**CRAS**	I (N.)	0	0	2	20	54	39	37	31	33	30
		R (N.)	0	0	1	17	47	34	37	29	30	22
		R (%)	.	.	50.0	85.0	87.0	87.2	100.0	93.5	90.9	73.3
**Calabria**	**MRSA**	I (N.)	0	0	89	198	57	28	82	153	180	217
		R (N.)	0	0	33	66	16	10	20	48	42	59
		R (%)	.	.	37.1	33.3	28.1	35.7	24.4	31.4	23.3	27.2
	**VRE-*faecium***	I (N.)	0	0	13	35	6	11	42	76	53	104
		R (N.)	0	0	3	3	2	6	13	22	19	41
		R (%)	.	.	23.1	8.6	33.3	54.5	31.0	28.9	35.8	39.4
	**3GCREC**	I (N.)	0	0	89	142	40	38	99	138	189	302
		R (N.)	0	0	33	61	16	20	31	55	70	125
		R (%)	.	.	37.1	43.0	40.0	52.6	31.3	39.9	37.0	41.4
	**CRKP**	I (N.)	0	0	50	127	26	27	58	103	175	287
		R (N.)	0	0	33	74	5	17	17	61	85	125
		R (%)	.	.	66.0	58.3	19.2	63.0	29.3	59.2	48.6	43.6
	**CRPA**	I (N.)	0	0	25	30	10	10	38	53	65	106
		R (N.)	0	0	13	7	3	7	5	13	9	20
		R (%)	.	.	52.0	23.3	30.0	70.0	13.2	24.5	13.8	18.9
	**CRAS**	I (N.)	0	0	26	53	13	4	82	107	58	63
		R (N.)	0	0	26	50	13	2	74	103	53	57
		R (%)	.	.	100.0	94.3	100.0	50.0	90.2	96.3	91.4	90.5
**Campania**	**MRSA**	I (N.)	528	484	539	714	723	815	771	859	919	992
		R (N.)	187	187	211	328	284	318	304	290	284	311
		R (%)	35.4	38.6	39.1	45.9	39.3	39.0	39.4	33.8	30.9	31.4
	**VRE-*faecium***	I (N.)	143	142	175	207	273	305	452	464	449	479
		R (N.)	2	3	12	20	31	29	59	125	114	121
		R (%)	1.4	2.1	6.9	9.7	11.4	9.5	13.1	26.9	25.4	25.3
	**3GCREC**	I (N.)	566	521	614	727	886	765	745	914	1142	1232

		R (N.)	252	222	273	343	393	302	309	349	414	468
		R (%)	44.5	42.6	44.5	47.2	44.4	39.5	41.5	38.2	36.3	38.0
	**CRKP**	I (N.)	360	367	311	476	486	552	522	646	920	907
		R (N.)	196	200	143	214	171	162	182	224	319	281
		R (%)	54.4	54.5	46.0	45.0	35.2	29.3	34.9	34.7	34.7	31.0
	**CRPA**	I (N.)	226	186	171	274	262	308	326	389	395	420
		R (N.)	78	59	48	77	46	62	93	111	98	69
		R (%)	34.5	31.7	28.1	28.1	17.6	20.1	28.5	28.5	24.8	16.4
	**CRAS**	I (N.)	314	272	337	448	359	630	558	511	449	441
		R (N.)	287	261	305	416	322	576	526	459	399	393
		R (%)	91.4	96.0	90.5	92.9	89.7	91.4	94.3	89.8	88.9	89.1
**Emilia-Romagna**	**MRSA**	I (N.)	519	554	584	1708	1693	1713	1787	2000	1982	2034
		R (N.)	155	149	165	518	488	434	412	478	432	382
		R (%)	29.9	26.9	28.3	30.3	28.8	25.3	23.1	23.9	21.8	18.8
	**VRE-*faecium***	I (N.)	106	143	113	490	457	518	597	671	698	686
		R (N.)	3	9	14	99	102	135	160	167	242	243
		R (%)	2.8	6.3	12.4	20.2	22.3	26.1	26.8	24.9	34.7	35.4
	**3GCREC**	I (N.)	1430	1457	1520	4334	4472	3784	4083	4638	4885	5239
		R (N.)	464	454	455	1172	1262	931	923	999	1125	1325
		R (%)	32.4	31.2	29.9	27.0	28.2	24.6	22.6	21.5	23.0	25.3
	**CRKP**	I (N.)	378	442	484	1091	1256	1120	1271	1525	1988	2284
		R (N.)	51	77	69	156	128	112	106	109	130	165
		R (%)	13.5	17.4	14.3	14.3	10.2	10.0	8.3	7.1	6.5	7.2
	**CRPA**	I (N.)	218	249	207	648	721	691	675	806	837	917
		R (N.)	26	24	14	84	67	78	67	83	110	77
		R (%)	11.9	9.6	6.8	13.0	9.3	11.3	9.9	10.3	13.1	8.4
	**CRAS**	I (N.)	31	48	41	43	44	35	32	33	200	154
		R (N.)	12	20	15	4	3	2	4	2	67	37
		R (%)	38.7	41.7	36.6	9.3	6.8	5.7	12.5	6.1	33.5	24.0
**Friuli Venezia Giulia**	**MRSA**	I (N.)	0	0	48	501	486	441	485	475	322	446
		R (N.)	0	0	11	126	143	102	120	99	59	89
		R (%)	.	.	22.9	25.1	29.4	23.1	24.7	20.8	18.3	20.0

	**VRE-*faecium***	I (N.)	0	0	25	95	84	115	122	115	105	105
		R (N.)	0	0	3	31	19	25	34	24	22	24
		R (%)	.	.	12.0	32.6	22.6	21.7	27.9	20.9	21.0	22.9
	**3GCREC**	I (N.)	0	0	92	1189	1270	1126	1051	1120	879	1272
		R (N.)	0	0	16	184	219	161	140	133	123	211
		R (%)	.	.	17.4	15.5	17.2	14.3	13.3	11.9	14.0	16.6
	**CRKP**	I (N.)	0	0	24	280	205	213	218	240	199	388
		R (N.)	0	0	3	25	21	15	28	15	16	27
		R (%)	.	.	12.5	8.9	10.2	7.0	12.8	6.3	8.0	7.0
	**CRPA**	I (N.)	0	0	13	152	156	160	152	157	148	209
		R (N.)	0	0	1	12	9	12	11	9	8	15
		R (%)	.	.	7.7	7.9	5.8	7.5	7.2	5.7	5.4	7.2
	**CRAS**	I (N.)	0	0	2	9	7	4	11	6	13	13
		R (N.)	0	0	0	7	2	2	7	0	10	3
		R (%)	.	.	.	77.8	28.6	50.0	63.6	.	76.9	23.1
**Lazio**	**MRSA**	I (N.)	292	345	192	451	771	974	938	1132	1574	1667
		R (N.)	123	156	92	230	393	461	378	517	595	589
		R (%)	42.1	45.2	47.9	51.0	51.0	47.3	40.3	45.7	37.8	35.3
	**VRE-*faecium***	I (N.)	54	60	72	102	165	406	405	520	576	692
		R (N.)	11	18	17	30	59	173	178	294	288	343
		R (%)	20.4	30.0	23.6	29.4	35.8	42.6	44.0	56.5	50.0	49.6
	**3GCREC**	I (N.)	266	334	319	457	757	1078	1127	1383	2073	2210
		R (N.)	86	128	99	155	338	331	354	440	751	811
		R (%)	32.3	38.3	31.0	33.9	44.6	30.7	31.4	31.8	36.2	36.7
	**CRKP**	I (N.)	202	272	244	332	678	771	778	1104	1485	1563
		R (N.)	96	94	80	121	270	300	238	462	565	567
		R (%)	47.5	34.6	32.8	36.4	39.8	38.9	30.6	41.8	38.00	36.3
	**CRPA**	I (N.)	97	123	112	183	291	439	468	576	703	773
		R (N.)	23	49	25	43	57	97	128	148	181	149
		R (%)	23.7	39.8	22.3	23.5	19.6	22.1	27.4	25.7	25.7	19.3
	**CRAS**	I (N.)	59	73	72	128	196	282	336	225	446	390
		R (N.)	38	52	54	98	159	232	307	207	372	319

		R (%)	64.4	71.2	75.0	76.6	81.1	82.3	91.4	92.0	83.4	81.8
**Liguria**	**MRSA**	I (N.)	76	78	98	91	506	748	768	887	802	904
		R (N.)	42	27	43	46	213	295	264	280	228	241
		R (%)	55.3	34.6	43.9	50.5	42.1	39.4	34.4	31.6	28.4	26.7
	**VRE-*faecium***	I (N.)	14	21	18	11	184	302	299	308	337	292
		R (N.)	2	4	1	4	88	93	130	99	113	65
		R (%)	14.3	19.0	5.6	36.4	47.8	30.8	43.5	32.1	33.5	22.3
	**3GCREC**	I (N.)	108	142	162	167	600	913	974	1157	1381	1576
		R (N.)	46	46	60	58	201	268	244	292	367	462
		R (%)	42.6	32.4	37.0	34.7	33.5	29.4	25.1	25.2	26.6	29.3
	**CRKP**	I (N.)	36	43	61	55	214	325	357	403	459	540
		R (N.)	18	20	27	17	56	62	70	60	47	61
		R (%)	50.0	46.5	44.3	30.9	26.2	19.1	19.6	14.9	10.2	11.3
	**CRPA**	I (N.)	10	17	21	17	146	214	184	239	255	242
		R (N.)	2	8	4	4	24	42	31	35	33	34
		R (%)	20.0	47.1	19.0	23.5	16.4	19.6	16.8	14.6	12.9	14.0
	**CRAS**	I (N.)	4	10	13	9	16	59	51	20	43	70
		R (N.)	2	6	10	8	4	8	28	13	19	21
		R (%)	50.0	60.0	76.9	88.9	25.0	13.6	54.9	65.0	44.2	30.0
**Lombardia**	**MRSA**	I (N.)	414	384	500	643	141	821	1347	2305	2423	2506
		R (N.)	135	119	163	190	49	230	380	624	651	696
		R (%)	32.6	31.0	32.6	29.5	34.8	28.0	28.2	27.1	26.9	27.8
	**VRE-*faecium***	I (N.)	116	133	214	241	129	460	652	935	889	943
		R (N.)	18	16	25	40	28	120	177	251	246	289
		R (%)	15.5	12.0	11.7	16.6	21.7	26.1	27.1	26.8	27.7	30.6
	**3GCREC**	I (N.)	726	787	1282	1485	625	2104	3550	5551	5396	5013
		R (N.)	178	185	305	370	163	435	758	1207	1276	1158
		R (%)	24.5	23.5	23.8	24.9	26.1	20.7	21.4	21.7	23.6	23.1
	**CRKP**	I (N.)	212	236	371	380	145	607	968	1625	1733	1716
		R (N.)	50	40	68	62	23	90	127	226	271	220
		R (%)	23.6	16.9	18.3	16.3	15.9	14.8	13.1	13.9	15.6	12.8
	**CRPA**	I (N.)	164	158	262	268	156	521	689	944	1014	899

		R (N.)	33	29	47	40	9	74	87	109	136	100
		R (%)	20.1	18.4	17.9	14.9	5.8	14.2	12.6	11.5	13.4	11.1
		I (N.)	39	44	60	39	7	15	194	203	280	257
	**CRAS**	R (N.)	17	12	29	14	0	0	162	165	115	122
		R (%)	43.6	27.3	48.3	35.9	.	.	83.5	81.3	41.1	47.5
		I (N.)	107	72	111	106	208	236	219	182	279	531
	**MRSA**	R (N.)	27	20	20	24	47	70	64	47	61	143
		R (%)	25.2	27.8	18.0	22.6	22.6	29.7	29.2	25.8	21.9	26.9
		I (N.)	21	27	31	29	73	94	99	94	128	193
	**VRE-*faecium***	R (N.)	0	5	7	4	15	39	38	32	54	86
		R (%)	.	18.5	22.6	13.8	20.5	41.5	38.4	34.0	42.2	44.6
		I (N.)	129	149	162	168	358	328	351	254	489	904
	**3GCREC**	R (N.)	38	50	60	53	109	100	106	56	136	279
**Marche**		R (%)	29.5	33.6	37.0	31.5	30.4	30.5	30.2	22.0	27.8	30.9
		I (N.)	52	50	96	75	91	187	214	176	315	495
	**CRKP**	R (N.)	18	10	30	16	26	70	73	75	114	192
		R (%)	34.6	20.0	31.3	21.3	28.6	37.4	34.1	42.6	36.2	38.8
		I (N.)	47	39	56	57	76	83	103	65	130	208
	**CRPA**	R (N.)	15	11	11	4	14	14	24	18	29	37
		R (%)	31.9	28.2	19.6	7.0	18.4	16.9	23.3	27.7	22.3	17.8
		I (N.)	18	12	15	22	29	48	74	48	113	119
	**CRAS**	R (N.)	13	8	6	13	25	40	59	43	101	104
		R (%)	72.2	66.7	40.0	59.1	86.2	83.3	79.7	89.6	89.4	87.4
		I (N.)	0	0	51	58	28	68	50	61	88	91
	**MRSA**	R (N.)	0	0	15	18	13	24	16	17	34	37
		R (%)	.	.	29.4	31.0	46.4	35.3	32.0	27.9	38.6	40.7
		I (N.)	0	0	8	4	7	8	9	16	13	21
**Molise**	**VRE-*faecium***	R (N.)	0	0	0	2	1	5	2	5	6	12
		R (%)	.	.	.	50.0	14.3	62.5	22.2	31.3	46.2	57.1
		I (N.)	0	0	47	84	39	52	64	68	98	132
	**3GCREC**	R (N.)	0	0	17	27	21	16	22	29	39	51
		R (%)	.	.	36.2	32.1	53.8	30.8	34.4	42.6	39.8	38.6

	**CRKP**	I (N.)	0	0	13	12	18	7	12	15	49	62
		R (N.)	0	0	2	3	7	2	3	2	10	19
		R (%)	.	.	15.4	25.0	38.9	28.6	25.0	13.3	20.4	30.6
	**CRPA**	I (N.)	0	0	6	8	16	12	8	14	22	24
		R (N.)	0	0	1	1	3	0	3	2	3	5
		R (%)	.	.	16.7	12.5	18.8	.	37.5	14.3	13.6	20.8
	**CRAS**	I (N.)	0	0	10	16	13	9	14	9	19	32
		R (N.)	0	0	8	15	10	8	14	9	18	31
		R (%)	.	.	80.0	93.8	76.9	88.9	100.0	100.0	94.7	96.9
**A.P. Bolzano**	**MRSA**	I (N.)	129	140	155	137	142	121	149	165	132	143
		R (N.)	19	20	15	10	23	8	11	9	11	7
		R (%)	14.7	14.3	9.7	7.3	16.2	6.6	7.4	5.5	8.3	4.9
	**VRE-*faecium***	I (N.)	23	26	28	30	28	28	49	41	36	36
		R (N.)	1	2	2	2	1	2	6	10	6	11
		R (%)	4.3	7.7	7.1	6.7	3.6	7.1	12.2	24.4	16.7	30.6
	**3GCREC**	I (N.)	300	314	339	390	397	350	378	376	420	429
		R (N.)	46	56	67	56	66	49	45	41	55	60
		R (%)	15.3	17.8	19.8	14.4	16.6	14.0	11.9	10.9	13.1	14.0
	**CRKP**	I (N.)	51	64	75	68	79	90	99	75	98	126
		R (N.)	0	4	4	2	3	3	5	1	1	1
		R (%)	.	6.3	5.3	2.9	3.8	3.3	5.1	1.3	1.0	0.8
	**CRPA**	I (N.)	24	18	37	29	34	38	48	52	39	41
		R (N.)	4	2	3	4	4	3	4	6	2	4
		R (%)	16.7	11.1	8.1	13.8	11.8	7.9	8.3	11.5	5.1	9.8
	**CRAS**	I (N.)	4	2	2	5	8	4	1	5	9	16
		R (N.)	0	0	0	0	0	0	0	0	0	0
		R (%)	.	.	.	.	.	.	.	.	.	.
**A.P. Trento**	**MRSA**	I (N.)	128	0	112	134	144	138	134	138	152	152
		R (N.)	40	0	22	33	34	19	23	20	22	16
		R (%)	31.3	.	19.6	24.6	23.6	13.8	17.2	14.5	14.5	10.5
	**VRE-*faecium***	I (N.)	56	35	28	38	41	39	43	36	48	47
		R (N.)	8	10	3	2	11	8	16	8	12	15

		R (%)	14.3	28.6	10.7	5.3	26.8	20.5	37.2	22.2	25.0	31.9
	**3GCREC**	I (N.)	357	382	300	377	373	302	342	399	458	454
		R (N.)	81	74	66	64	70	59	50	86	96	91
		R (%)	22.7	19.4	22.0	17.0	18.8	19.5	14.6	21.6	21.0	20.0
	**CRKP**	I (N.)	79	68	51	81	59	65	85	77	86	105
		R (N.)	1	2	1	2	3	2	7	2	3	1
		R (%)	1.3	2.9	2.0	2.5	5.1	3.1	8.2	2.6	3.5	1.0
	**CRPA**	I (N.)	0	0	42	39	62	38	57	56	64	57
		R (N.)	0	0	7	5	5	1	8	6	6	5
		R (%)	.	.	16.7	12.8	8.1	2.6	14.0	10.7	9.4	8.8
	**CRAS**	I (N.)	1	6	6	6	0	2	3	4	3	6
		R (N.)	1	0	0	2	0	0	0	0	0	0
		R (%)	100.0	.	.	33.3	.	.	.	.	.	.
**Piemonte**	**MRSA**	I (N.)	382	337	426	455	358	586	684	765	843	814
		R (N.)	170	145	178	161	118	206	225	307	212	212
		R (%)	44.5	43.0	41.8	35.4	33.0	35.2	32.9	40.1	25.1	26.0
	**VRE-*faecium***	I (N.)	84	96	88	78	58	144	196	242	297	264
		R (N.)	21	29	32	18	17	43	80	102	125	95
		R (%)	25.0	30.2	36.4	23.1	29.3	29.9	40.8	42.1	42.1	36.0
	**3GCREC**	I (N.)	758	735	918	935	574	991	1216	1461	1642	1664
		R (N.)	195	198	263	260	165	216	235	353	450	446
		R (%)	25.7	26.9	28.6	27.8	28.7	21.8	19.3	24.2	27.4	26.8
	**CRKP**	I (N.)	278	273	301	336	251	405	435	552	664	689
		R (N.)	126	143	123	116	70	134	138	130	252	173
		R (%)	45.3	52.4	40.9	34.5	27.9	33.1	31.7	23.6	38.0	25.1
	**CRPA**	I (N.)	123	128	162	158	139	228	170	326	345	340
		R (N.)	29	26	36	19	21	40	28	48	37	40
		R (%)	23.6	20.3	22.2	12.0	15.1	17.5	16.5	14.7	10.7	11.8
	**CRAS**	I (N.)	60	58	48	41	15	72	117	114	60	51
		R (N.)	40	44	28	23	7	57	100	91	31	18
		R (%)	66.7	75.9	58.3	56.1	46.7	79.2	85.5	79.8	51.7	35.3
**Puglia**	**MRSA**	I (N.)	34	96	90	184	183	362	405	409	523	775

		R (N.)	5	30	31	58	59	129	136	110	136	201
		R (%)	14.7	31.3	34.4	31.5	32.2	35.6	33.6	26.9	26.0	25.9
	**VRE-*faecium***	I (N.)	2	54	48	61	58	154	221	237	229	326
		R (N.)	0	0	2	7	7	17	29	62	63	92
		R (%)	.	.	4.2	11.5	12.1	11.0	13.1	26.2	27.5	28.2
	**3GCREC**	I (N.)	57	56	52	320	318	582	513	661	883	1093
		R (N.)	22	21	23	150	142	193	196	231	319	403
		R (%)	38.6	37.5	44.2	46.9	44.7	33.2	38.2	34.9	36.1	36.9
	**CRKP**	I (N.)	27	106	139	174	227	417	504	507	660	777
		R (N.)	16	73	79	92	111	237	289	254	280	319
		R (%)	59.3	68.9	56.8	52.9	48.9	56.8	57.3	50.1	42.4	41.1
	**CRPA**	I (N.)	9	58	76	67	91	179	197	236	283	439
		R (N.)	3	25	23	19	33	46	51	60	56	77
		R (%)	33.3	43.1	30.3	28.4	36.3	25.7	25.9	25.4	19.8	17.5
	**CRAS**	I (N.)	14	80	85	121	125	295	484	288	196	436
		R (N.)	12	67	69	106	110	273	459	266	174	309
		R (%)	85.7	83.8	81.2	87.6	88.0	92.5	94.8	92.4	88.8	70.9
**Sardegna**	**MRSA**	I (N.)	97	98	142	99	70	0	101	76	246	335
		R (N.)	20	24	45	32	16	0	18	19	51	63
		R (%)	20.6	24.5	31.7	32.3	22.9	.	17.8	25.0	20.7	18.8
	**VRE-*faecium***	I (N.)	17	30	28	31	31	0	30	66	115	167
		R (N.)	1	0	0	1	0	0	1	2	12	16
		R (%)	5.9	.	.	3.2	.	.	3.3	3.0	10.4	9.6
	**3GCREC**	I (N.)	176	186	176	169	171	0	145	250	355	697
		R (N.)	33	42	34	28	38	0	32	39	47	114
		R (%)	18.8	22.6	19.3	16.6	22.2	.	22.1	15.6	13.2	16.4
	**CRKP**	I (N.)	47	67	66	62	85	0	5	137	296	427
		R (N.)	9	23	19	16	16	0	2	34	66	78
		R (%)	19.1	34.3	28.8	25.8	18.8	.	40.0	24.8	22.3	18.3
	**CRPA**	I (N.)	31	21	49	39	49	0	36	92	144	201
		R (N.)	5	3	11	10	11	0	9	16	19	21
		R (%)	16.1	14.3	22.4	25.6	22.4	.	25.0	17.4	13.2	10.4

		I (N.)	19	14	10	7	11	0	10	16	37	36
	**CRAS**	R (N.)	14	11	8	5	8	0	10	13	30	22
		R (%)	73.7	78.6	80.0	71.4	72.7	.	100.0	81.3	81.1	61.1
		I (N.)	23	19	0	112	624	524	528	673	825	777
	**MRSA**	R (N.)	5	10	0	43	290	242	242	242	284	225
		R (%)	21.7	52.6	.	38.4	46.5	46.2	45.8	36.0	34.4	29.0
		I (N.)	7	6	13	27	156	248	389	444	463	451
	**VRE-*faecium***	R (N.)	0	1	1	0	11	43	81	127	142	193
		R (%)	.	16.7	7.7	.	7.1	17.3	20.8	28.6	30.7	42.8
		I (N.)	41	31	31	102	922	646	668	809	1179	1471
	**3GCREC**	R (N.)	17	8	11	36	418	289	252	284	461	581
		R (%)	41.5	25.8	35.5	35.3	45.3	44.7	37.7	35.1	39.1	39.5
**Sicilia**		I (N.)	26	17	29	132	1065	988	971	1047	1167	1252
	**CRKP**	R (N.)	3	1	7	57	563	594	582	508	681	700
		R (%)	11.5	5.9	24.1	43.2	52.9	60.1	59.9	48.5	58.4	55.9
		I (N.)	10	4	5	54	350	310	291	347	557	571
	**CRPA**	R (N.)	4	1	1	14	68	77	88	77	88	100
		R (%)	40.0	25.0	20.0	25.9	19.4	24.8	30.2	22.2	15.8	17.5
		I (N.)	23	8	11	47	306	494	629	596	479	624
	**CRAS**	R (N.)	23	6	11	45	280	423	555	561	438	593
		R (%)	100.0	75.0	100.0	95.7	91.5	85.6	88.2	94.1	91.4	95.0
		I (N.)	98	212	224	837	1313	1145	1413	1458	1359	1436
	**MRSA**	R (N.)	20	57	70	240	372	333	328	356	241	277
		R (%)	20.4	26.9	31.3	28.7	28.3	29.1	23.2	24.4	17.7	19.3
		I (N.)	82	93	76	251	390	390	566	564	578	520
	**VRE-*faecium***	R (N.)	16	21	21	71	62	37	97	161	168	186
**Toscana**		R (%)	19.5	22.6	27.6	28.3	15.9	9.5	17.1	28.5	29.1	35.8
		I (N.)	352	412	379	1750	2492	2001	2374	2402	2496	2608
	**3GCREC**	R (N.)	130	158	126	624	815	560	618	604	696	709
		R (%)	36.9	38.3	33.2	35.7	32.7	28.0	26.0	25.1	27.9	27.2
	**CRKP**	I (N.)	159	175	162	714	1178	1076	1271	1451	1493	1692
		R (N.)	57	63	64	220	333	276	226	244	283	312

		R (%)	35.8	36.0	39.5	30.8	28.3	25.7	17.8	16.8	19.0	18.4
	**CRPA**	I (N.)	77	108	81	367	506	560	619	716	625	769
		R (N.)	21	18	14	44	39	69	72	96	93	100
		R (%)	27.3	16.7	17.3	12.0	7.7	12.3	11.6	13.4	14.9	13.0
	**CRAS**	I (N.)	46	41	61	161	172	180	276	170	241	223
		R (N.)	34	38	56	117	111	119	226	149	152	140
		R (%)	73.9	92.7	91.8	72.7	64.5	66.1	81.9	87.6	63.1	62.8
**Umbria**	**MRSA**	I (N.)	0	99	95	200	396	447	478	555	509	482
		R (N.)	0	25	23	65	143	181	203	258	194	182
		R (%)	.	25.3	24.2	32.5	36.1	40.5	42.5	46.5	38.1	37.8
	**VRE-*faecium***	I (N.)	0	31	42	65	102	162	190	207	197	204
		R (N.)	0	7	10	31	38	80	103	145	134	143
		R (%)	.	22.6	23.8	47.7	37.3	49.4	54.2	70.0	68.0	70.1
	**3GCREC**	I (N.)	0	144	193	302	586	601	593	657	716	725
		R (N.)	0	37	49	100	178	177	179	182	183	187
		R (%)	.	25.7	25.4	33.1	30.4	29.5	30.2	27.7	25.6	25.8
	**CRKP**	I (N.)	0	56	46	133	198	220	238	308	366	343
		R (N.)	0	20	8	56	72	87	109	130	165	125
		R (%)	.	35.7	17.4	42.1	36.4	39.5	45.8	42.2	45.1	36.4
	**CRPA**	I (N.)	0	39	30	50	111	122	164	166	141	163
		R (N.)	0	11	6	10	20	26	44	41	26	32
		R (%)	.	28.2	20.0	20.0	18.0	21.3	26.8	24.7	18.4	19.6
	**CRAS**	I (N.)	0	7	28	31	62	80	65	77	147	107
		R (N.)	0	5	25	24	47	63	56	74	127	92
		R (%)	.	71.4	89.3	77.4	75.8	78.8	86.2	96.1	86.4	86.0
**Valle D'Aosta**	**MRSA**	I (N.)	0	0	0	56	21	54	59	62	72	68
		R (N.)	0	0	0	23	7	17	16	17	14	16
		R (%)	.	.	.	41.1	33.3	31.5	27.1	27.4	19.4	23.5
	**VRE-*faecium***	I (N.)	0	0	0	9	6	12	13	23	23	19
		R (N.)	0	0	0	1	0	1	2	4	7	8
		R (%)	.	.	.	11.1	.	8.3	15.4	17.4	30.4	42.1
	**3GCREC**	I (N.)	0	0	0	139	60	110	112	125	157	147

		R (N.)	0	0	0	61	31	39	35	32	51	33
		R (%)	.	.	.	43.9	51.7	35.5	31.3	25.6	32.5	22.4
	**CRKP**	I (N.)	0	0	0	35	18	32	24	19	26	47
		R (N.)	0	0	0	9	6	9	13	1	8	15
		R (%)	.	.	.	25.7	33.3	28.1	54.2	5.3	30.8	31.9
	**CRPA**	I (N.)	0	0	0	8	2	9	20	14	17	19
		R (N.)	0	0	0	0	0	2	1	0	2	0
		R (%)	.	.	.	.	.	22.2	5.0	.	11.8	.
	**CRAS**	I (N.)	0	0	0	2	1	0	2	0	1	7
		R (N.)	0	0	0	1	0	0	0	0	0	4
		R (%)	.	.	.	50.0	.	.	.	.	.	57.1
**Veneto**	**MRSA**	I (N.)	210	100	133	1465	1554	1526	1684	1475	1447	1616
		R (N.)	79	45	67	549	538	501	508	378	338	343
		R (%)	37.6	45.0	50.4	37.5	34.6	32.8	30.2	25.6	23.4	21.2
	**VRE-*faecium***	I (N.)	42	47	38	431	506	675	768	676	626	619
		R (N.)	2	1	1	47	81	102	177	131	122	152
		R (%)	4.8	2.1	2.6	10.9	16.0	15.1	23.0	19.4	19.5	24.6
	**3GCREC**	I (N.)	327	291	337	2739	3139	2723	3142	2844	3167	3858
		R (N.)	94	91	108	778	936	717	723	664	775	1030
		R (%)	28.7	31.3	32.0	28.4	29.8	26.3	23.0	23.3	24.5	26.7
	**CRKP**	I (N.)	92	68	92	999	997	1037	1193	1103	1245	1332
		R (N.)	23	9	14	234	181	234	267	209	221	150
		R (%)	25.0	13.2	15.2	23.4	18.2	22.6	22.4	18.9	17.8	11.3
	**CRPA**	I (N.)	46	58	65	507	594	625	716	602	721	788
		R (N.)	4	15	12	69	80	77	102	87	93	95
		R (%)	8.7	25.9	18.5	13.6	13.5	12.3	14.2	14.5	12.9	12.1
	**CRAS**	I (N.)	33	31	40	119	150	213	209	165	122	110
		R (N.)	27	24	32	79	111	145	172	133	67	56
		R (%)	81.8	77.4	80.0	66.4	74.0	68.1	82.3	80.6	54.9	50.9
**Italy**	**MRSA**	I (N.)	3037	3018	3634	8323	9682	10976	12191	14083	14943	16226
		R (N.)	1027	1014	1219	2823	3322	3673	3716	4206	3969	4163
		R (%)	33.8	33.6	33.5	33.9	34.3	33.5	30.5	29.9	26.6	25.7

	**VRE-*faecium***	I (N.)	767	944	1063	2291	2839	4177	5202	5910	6022	6323
		R (N.)	85	126	154	434	605	984	1399	1815	1958	2206
		R (%)	11.1	13.3	14.5	18.9	21.3	23.6	26.9	30.7	32.5	34.9
	**3GCREC**	I (N.)	5593	5941	7079	16253	18410	18760	21920	25683	28581	31649
		R (N.)	1682	1770	2089	4664	5682	4955	5341	6223	7625	8775
		R (%)	30.1	29.8	29.5	28.7	30.9	26.4	24.4	24.2	26.7	27.7
	**CRKP**	I (N.)	1999	2304	2633	5661	7325	8282	9297	11248	13664	15331
		R (N.)	664	779	778	1516	2086	2447	2524	2801	3615	3673
		R (%)	33.2	33.8	29.5	26.8	28.5	29.5	27.1	24.9	26.5	24.0
	**CRPA**	I (N.)	1082	1206	1433	3014	3794	4616	5047	5972	6645	7374
		R (N.)	247	281	281	476	521	736	866	981	1065	1014
		R (%)	22.8	23.3	19.6	15.8	13.7	15.9	17.2	16.4	16.0	13.8
	**CRAS**	I (N.)	665	706	869	1383	1588	2550	3297	2744	3103	3319
		R (N.)	520	554	683	1096	1259	2064	2905	2429	2353	2465
		R (%)	78.2	78.5	78.6	79.2	79.3	80.9	88.1	88.5	75.8	74.3

I (N.) = number of isolates; R (N.) = number of resistant isolates; R (%) = percentage of resistance.
MRSA = *Staphylococcus aureus* resistant to methicillin; VRE-*faecium* = *Enterococcus faecium* resistant to vancomycin; 3GCREC = *Escherichia coli* resistant to third-generation cephalosporins; CRKP = *Klebsiella pneumoniae* resistant to carbapenems; CRPA = *Pseudomonas aeruginosa* resistant to carbapenems; CRAS = *Acinetobacter species* resistant to carbapenems

Supplementary Table S2. Linear regression models of antimicrobial-resistance in isolated species in Italy, by region and species, from 2015 to 2024.

Region	MRSA			VRE-*faecium*			3GCREC			CRKP			CRPA			CRAS		
	β	95%CI	p-value	β	95%CI	p-value	β	95%CI	p-value	β	95%CI	p-value	β	95%CI	p-value	β	95%CI	p-value
Abruzzo	-0.52 c	-10.39; 9.35	0.8973	1.35 c	-4.86; 7.56	0.6005	0.72 c	-1.72; 3.16	0.4819	6.19 c	2.46; 9.92	**0.008**	2.86 c	-0.04; 5.75	0.0521	6.84 c	-10.59; 24.28	0.3593
Basilicata	-0.18 b	-1.73; 1.38	0.7909	2.09 b	-1.58; 5.76	0.2133	0.72 b	-0.87; 2.31	0.3081	2.64 b	-1.90; 7.17	0.2048	-2.87 b	-8.40; 2.67	0.2522	2.68 b	-3.05; 8.40	0.2962
Calabria	-1.44 b	-2.95; 0.08	0.0595	2.54 b	-2.19; 7.27	0.2371	-0.26 b	-2.74; 2.23	0.8093	-1.42 b	-8.12; 5.29	0.6238	-4.2 b	-11.23; 2.84	0.1947	-0.62 b	-7.23; 5.99	0.8266
Campania	-0.83	-1.83; 0.18	0.0951	2.95	2.06; 3.83	**0.0001**	-0.95	-1.51; -0.38	**0.005**	-2.68	-3.91; -1.45	**0.001**	-1.25	-2.50; 0.01	0.052	-0.41	-0.95; 0.12	0.1139
Emilia-Romagna	-1.11	-1.59; -0.63	**0.0007**	3.5	2.70; 4.31	**<0.0001**	-1.09	-1.59; -0.59	**0.001**	-1.13	-1.55; -0.72	**0.0002**	0.02	-0.52; 0.56	0.936	-2.02	-5.70; 1.65	0.2403
Friuli Venezia Giulia	-0.93 b	-2.00; 0.13	0.0749	0.23 b	-2.18; 2.64	0.8224	-0.36 b	-1.08; 0.37	0.2726	-0.58 b	-1.43; 0.26	0.1433	-0.2 b	-0.55; 0.16	0.2242	1.01 b	-11.90; 13.93	0.8543
Lazio	-0.97	-2.18; 0.24	0.1017	3.74	2.54; 4.95	**0.0001**	0.05	-1.13; 1.22	0.9313	-0.3	-1.57; 0.96	0.5938	-0.65	-2.13; 0.83	0.3418	2.26	0.89; 3.62	**0.0052**
Liguria	-2.5	-3.99; -1.02	**0.0045**	1.88	-1.31; 5.07	0.2116	-1.53	-2.38; -0.68	**0.0032**	-4.79	-5.78; -3.80	**<0.0001**	-2.01	-4.12; 0.09	0.0585	-2.81	-8.70; 3.08	0.3037
Lombardia	-0.67	-1.17; -0.17	**0.0151**	2.16	1.46; 2.87	**0.0001**	-0.23	-0.64; 0.18	0.2267	-0.84	-1.33; -0.35	**0.0042**	-0.89	-1.75; -0.03	**0.0445**	2.66	-4.59; 9.91	0.4215
Marche	0.24	-0.73; 1.21	0.5818	4.36	2.57; 6.15	**0.0005**	-0.65	-1.54; 0.25	0.1329	1.54	0.02; 3.07	**0.0475**	-0.49	-2.36; 1.38	0.5649	3.65	0.49; 6.82	**0.0288**
Molise	0.69 b	-1.80; was 3.19	0.5213	4.66 b	-3.07; 12.39	0.1905	0.3 b	-2.67; 3.27	0.8123	2.41	-0.40; 5.22	0.0835	0.69 b	-3.52; 4.90	0.701	10.09	3.44; 16.75	**0.0081**
A.P. Bolzano	-0.97	-1.74; -0.20	**0.0193**	2.46	1.05; 3.87	**0.0038**	-0.6	-1.14; -0.07	**0.0322**	-0.27	-0.81; 0.27	0.2901	-0.65	-1.37; 0.07	0.0703	NA	NA	NA
A.P. Trento	-0.87	-3.07; 1.34	0.391	1.7	-0.56; 3.96	0.1216	-0.13	-0.78; 0.52	0.6549	0.12	-0.44; 0.67	0.6355	-0.63 b	-2.26; 0.99	0.3784	-6.06	-13.19; 1.07	0.0855
Piemonte	-1.85	-2.86; -0.84	**0.0029**	1.6	0.25; 2.95	**0.0254**	-0.25	-1.05; 0.56	0.4975	-2.26	-3.87; -0.64	**0.0123**	-1.18	-1.90; -0.46	**0.0053**	-1.36	-5.67; 2.95	0.4888
Puglia	0.22	-1.43; 1.87	0.7691	3.36	2.52; 4.19	**<0.0001**	-0.66	-1.77; 0.44	0.2052	-2.19	-3.53; -0.86	**0.0053**	-2.11	-3.22; -0.99	**0.0024**	-0.1	-1.95; 1.76	0.9064
Sardegna	-0.86	-3.18; 1.45	0.4146	0.74	-0.14; 1.61	0.0888	-0.68	-2.38; 1.03	0.3866	-0.53	-3.40; 2.34	0.6816	-0.66	-2.69; 1.38	0.4791	-0.46	-7.59; 6.67	0.8848
Sicilia	0.85	-3.24; 4.94	0.6446	4	2.11; 5.89	**0.0012**	0.48	-0.98; 1.95	0.4682	5.74	2.74; 8.73	**0.0022**	-1.44	-2.94; 0.06	0.0575	0.07	-1.93; 2.08	0.9359
Toscana	-0.75	-1.85; 0.34	0.15	0.95	-1.03; 2.93	0.2999	-1.42	-2.02; -0.82	**0.0006**	-2.61	-3.49; -1.73	**0.0001**	-0.95	-2.13; 0.22	0.0975	-1.81	-4.67; 1.04	0.1817
Umbria	2.1 a	0.55; 3.66	**0.0151**	6.40 a	4.45; 8.35	**0.0001**	-0.17 a	-1.06; 0.72	0.6718	1.59 a	-0.80; 3.99	0.1599	-0.35 a	-1.53; 0.83	0.5056	1.63 a	-0.44; 3.69	0.1045
Valle d'Aosta	-3.03 c	-4.46; -1.59	**0.0029**	5.82 c	2.32; 9.32	**0.0079**	-4.03 c	-6.86; -1.19	**0.0147**	-0.33 c	-7.95; 7.29	0.916	1.19 c	-5.26; 7.65	0.6347	0.76 c	-13.16; 14.68	0.8938
Veneto	-2.71	-3.89; -1.52	**0.0008**	2.54	1.69; 3.40	**0.0001**	-0.78	-1.35; -0.21	**0.0138**	-0.42	-1.60; 0.75	0.4331	-0.48	-1.67; 0.70	0.3754	-2.37	-4.73; 0.00	**0.0496**
**Italy**	-0.91	-1.35; -0.48	**0.0013**	2.76	2.58; 2.94	**<0.0001**	-0.53	-0.99; -0.06	**0.0304**	-0.94	-1.35; -0.53	**0.0008**	-0.86	-1.44; -0.27	**0.0095**	0.14	-1.11; 1.40	0.7977

*In bold p-value <0.05. The starting year for the regression model was 2015 unless otherwise specified. [a]= from 2016; [b]=from 2017 [c]= from 2018.
MRSA = *Staphylococcus aureus* resistant to methicillin; VRE-*faecium* = *Enterococcus faecium* resistant to vancomycin; 3GCREC = *Escherichia coli* resistant to third-generation cephalosporins; CRKP = *Klebsiella pneumoniae* resistant to carbapenems; CRPA = *Pseudomonas aeruginosa* resistant to carbapenems; CRAS = *Acinetobacter species* resistant to carbapenems.
NA: Data not available

Supplementary Figure S1. Linear regression models of MRSA resistance in Italy, by region.

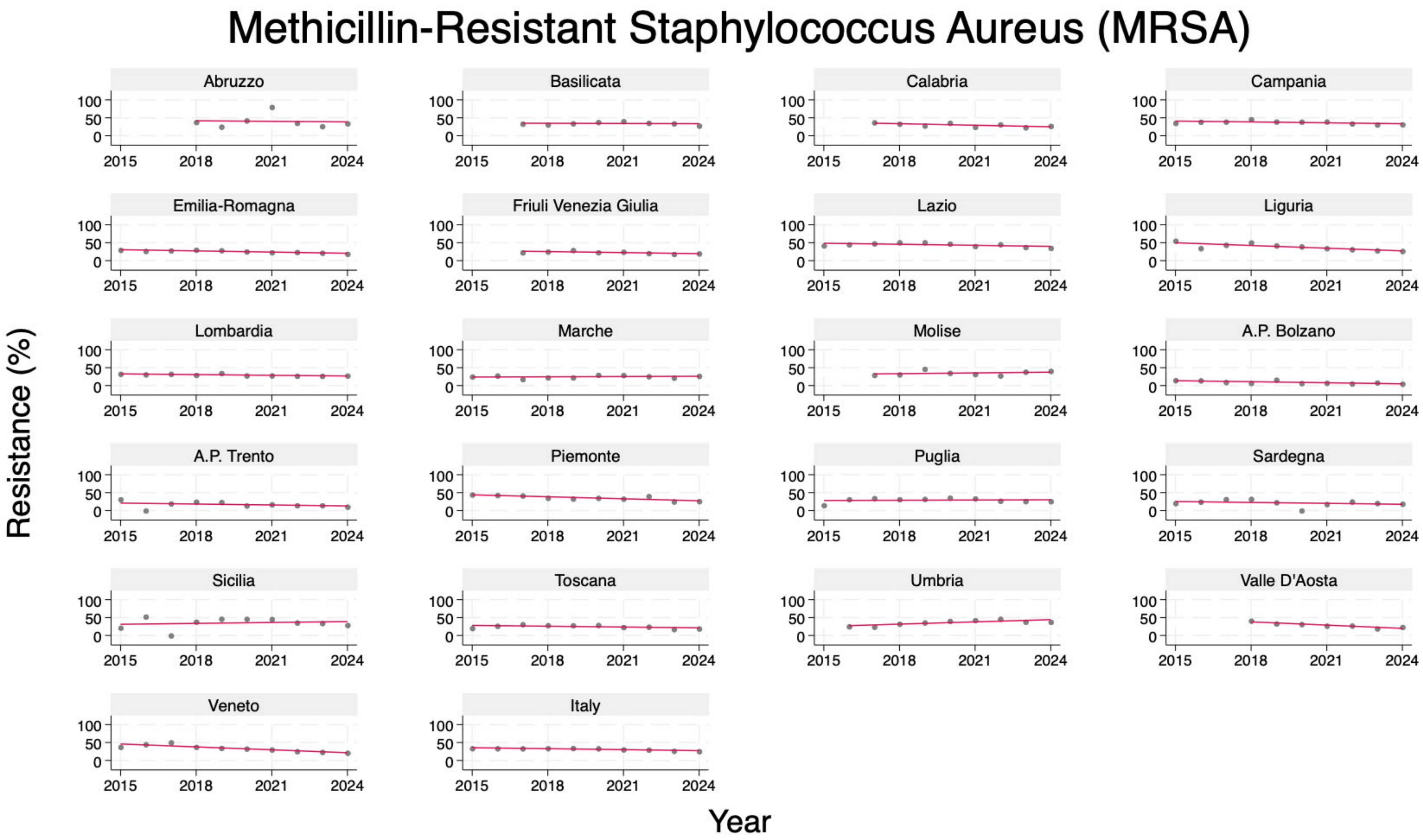

Supplementary Figure S2. Linear regression models of VRE-*faecium* resistance in Italy, by region.

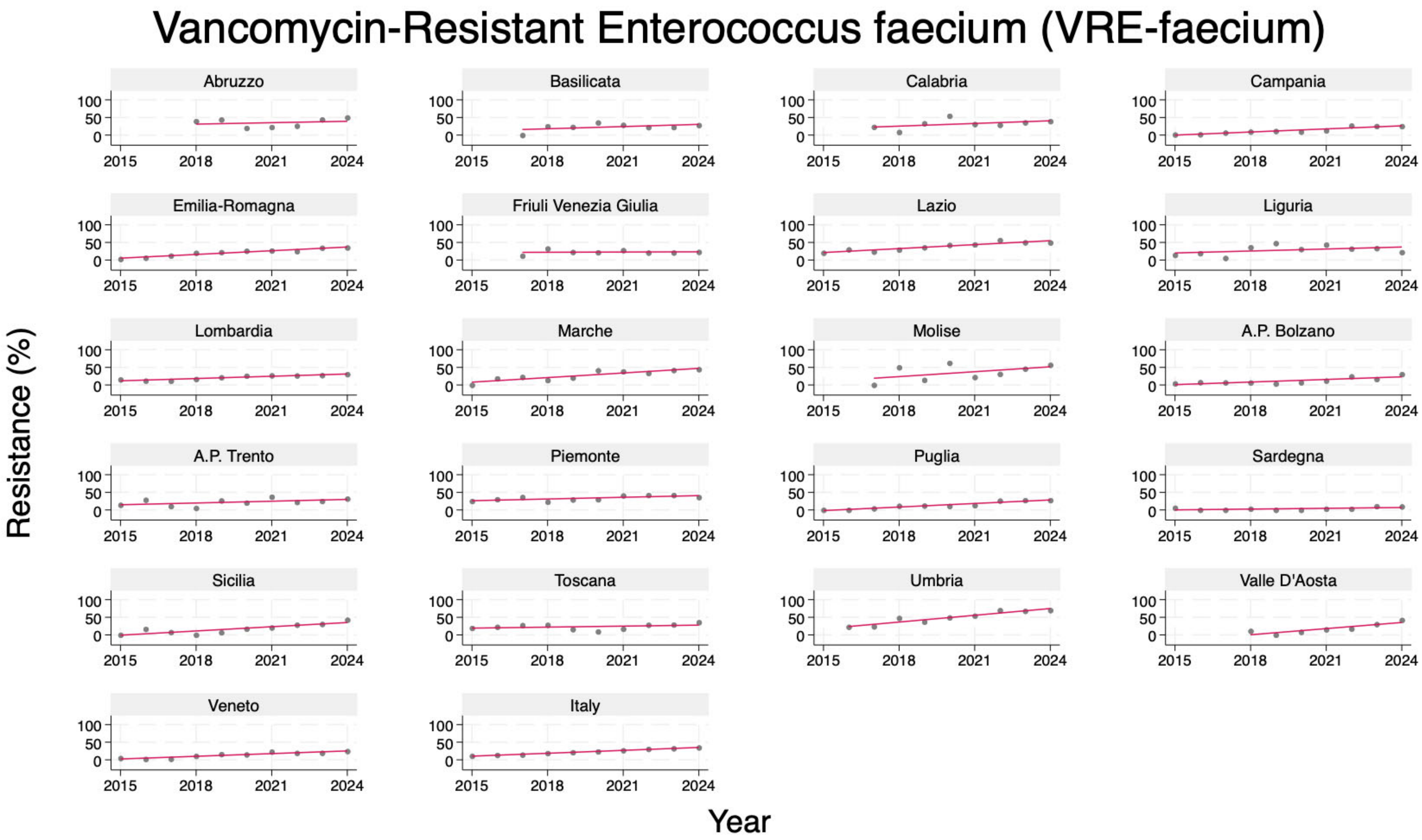

Supplementary Figure S3. Linear regression models of 3GCREC resistance in Italy, by region.

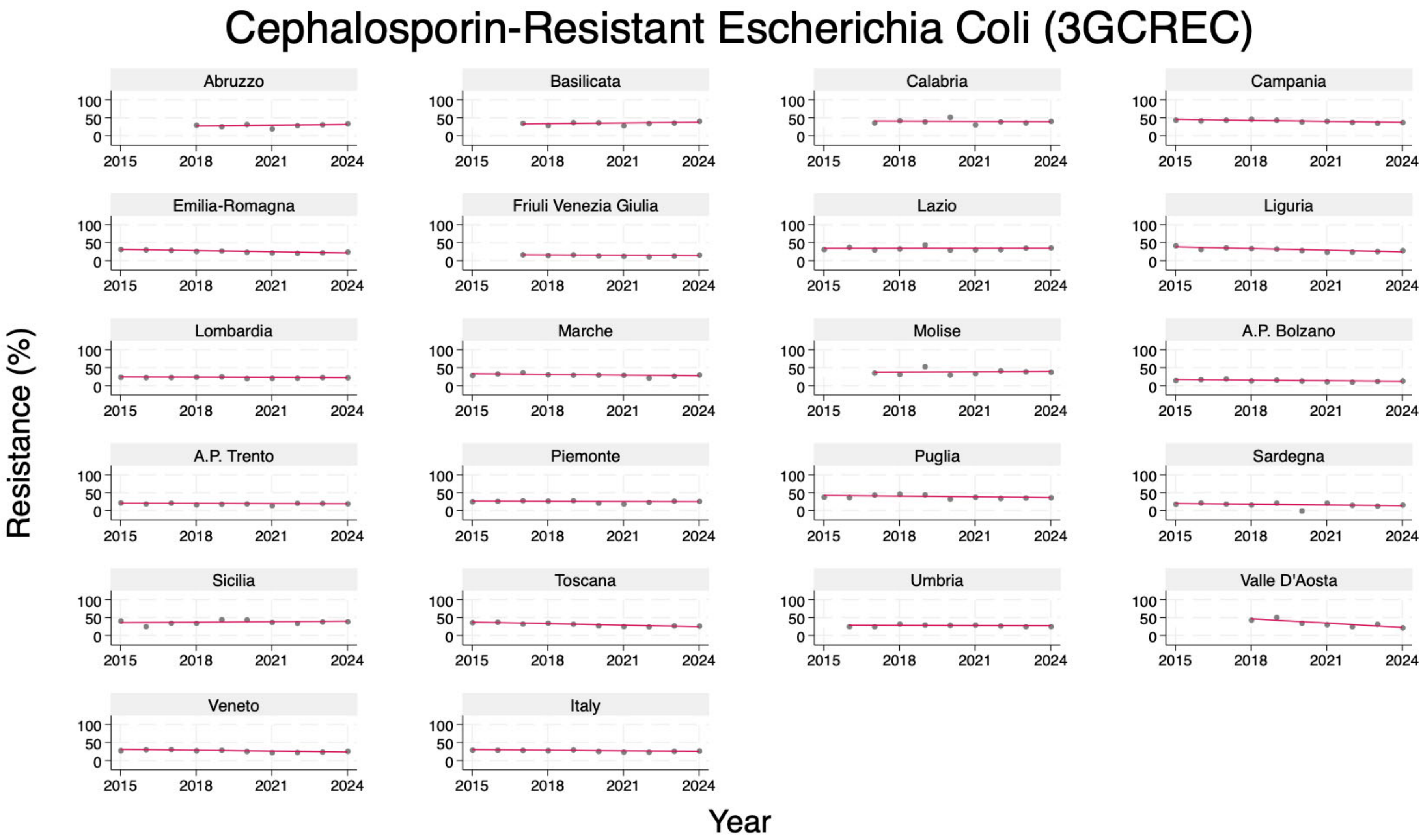

Supplementary Figure S4. Linear regression models of CRKP resistance in Italy, by region.

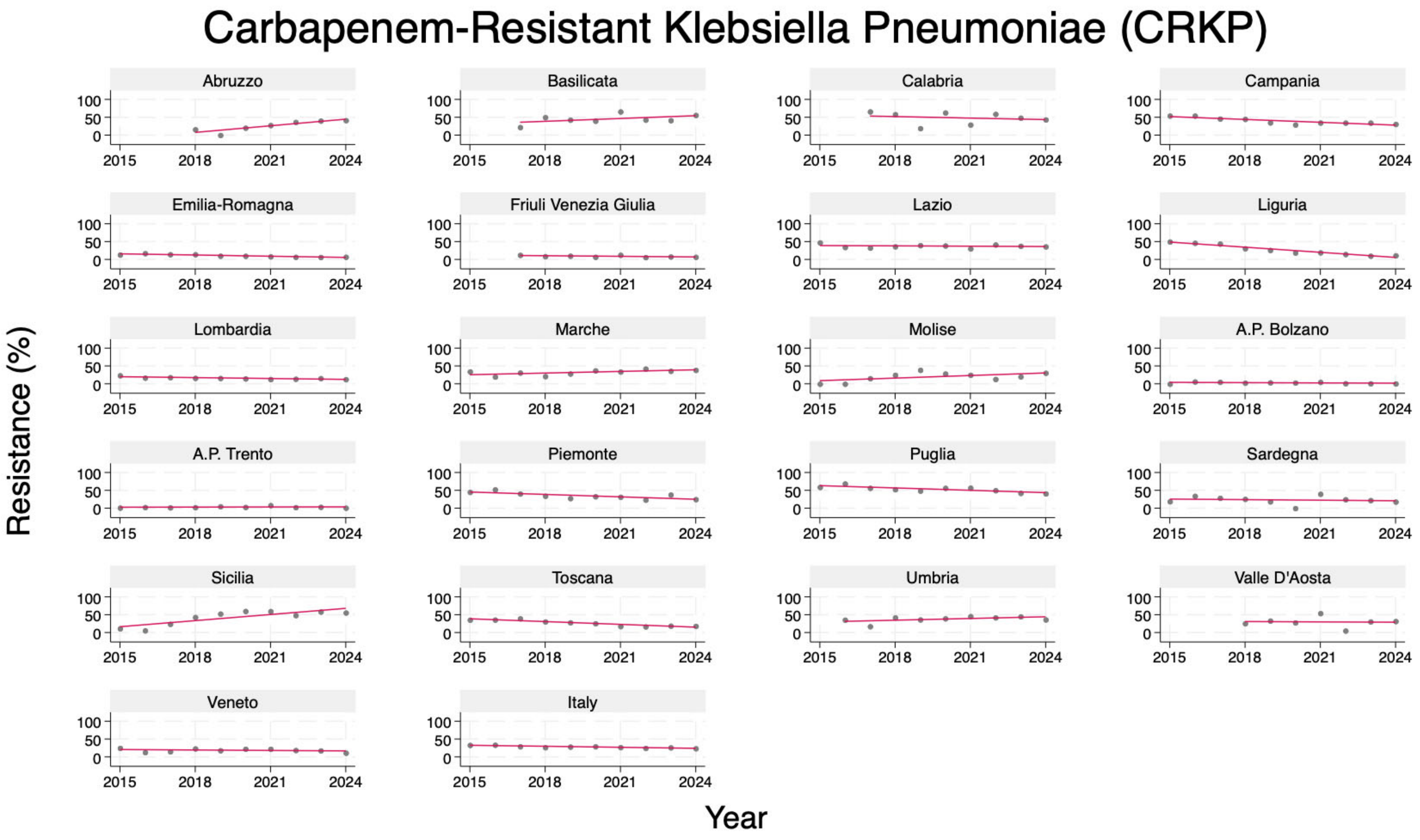

Supplementary Figure S5. Linear regression models of CRPA resistance in Italy, by region.

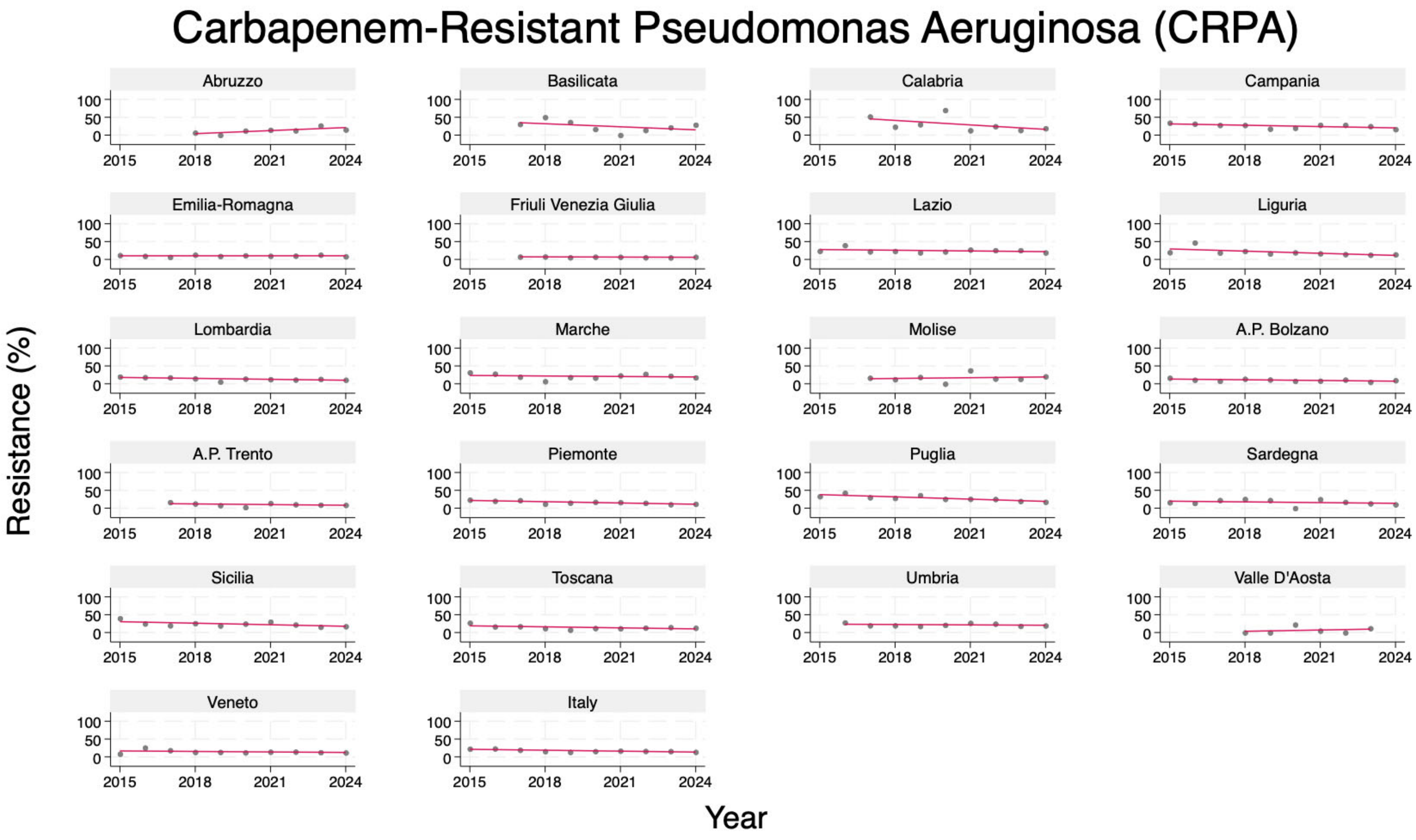

Supplementary Figure S6. Linear regression models of CRAS resistance in Italy, by region.

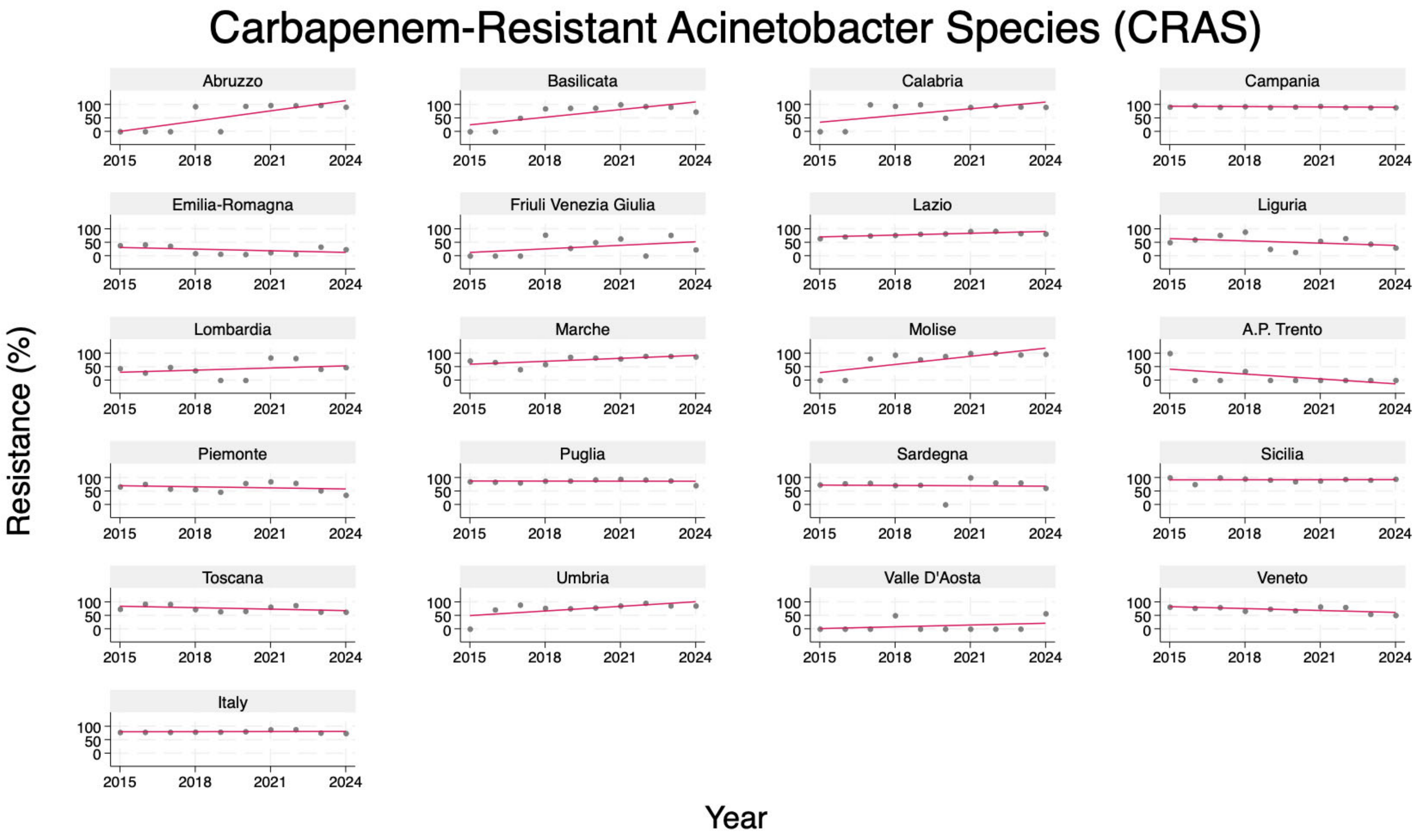

Supplementary File S1. STROBE Statement. checklist of items that should be included in reports of observational studies

	Item No.	Recommendation	Page No.	Relevant text from manuscript
**Title and abstract**	1	(*a*) Indicate the study's design with a commonly used term in the title or the abstract	1	Abstract
		(*b*) Provide in the abstract an informative and balanced summary of what was done and what was found	1	Abstract
**Introduction**				
Background/rationale	2	Explain the scientific background and rationale for the investigation being reported	2, 3	Introduction
Objectives	3	State specific objectives, including any prespecified hypotheses	3	"[…] this study systematically consolidates AR-ISS data from 2015 to 2024 and applies a consistent analytical framework to six priority pathogen–resistance combinations."
**Methods**				
Study design	4	Present key elements of study design early in the paper	10	"We conducted an observational ecological time-series analysis based on nationally aggregated data, reported in accordance with the STROBE guidelines"
Setting	5	Describe the setting, locations, and relevant dates, including periods of recruitment, exposure, follow-up, and data collection	10	"We considered the period from 2015 (or the first available year for each region) through 2024."
Participants	6	(*a*) *Cohort study*—Give the eligibility criteria, and the sources and methods of selection of participants. Describe methods of follow-up	Not applicable	
		Case-control study—Give the eligibility criteria, and the sources and methods of case ascertainment and control selection. Give the rationale for the choice of cases and controls	Not applicable	
			11	

		Cross-sectional study—Give the eligibility criteria, and the sources and methods of selection of participants		"We included AMR data presented as the percentage of resistant isolates relative to the total number of isolates identified. We collected data for the six priority organisms and their relative resistance rates: Methicillin-Resistant *Staphylococcus aureus* (MRSA), Vancomycin-Resistant *Enterococcus faecium* (VRE-*faecium*), third-generation Cephalosporin-Resistant *Escherichia coli* (3GCREC), Carbapenem-Resistant *Klebsiella pneumoniae* (CRKP), Carbapenem-Resistant *Pseudomonas aeruginosa* (CRPA), and Carbapenem-Resistant *Acinetobacter species* (CRAS) are recognized among the most critical bacteria."
		(*b*) *Cohort study*—For matched studies, give matching criteria and number of exposed and unexposed<br>*Case-control study*—For matched studies, give matching criteria and the number of controls per case	Not applicable	
Variables	7	Clearly define all outcomes, exposures, predictors, potential confounders, and effect modifiers. Give diagnostic criteria, if applicable	Not applicable	
Data sources/ measurement	8*	For each variable of interest, give sources of data and details of methods of assessment (measurement). Describe comparability of assessment methods if there is more than one group	10	"Data on resistance rates were extracted from the AR-ISS (Antimicrobial Resistance - National Institute of Health) surveillance system, specifically from the annual reports "*AR-ISS: Sorveglianza Nazionale dell'Antibiotico-Resistenza*."
Bias	9	Describe any efforts to address potential sources of bias	10	"Participating laboratories, recruited through regional health authorities, routinely perform antimicrobial susceptibility testing (AST) as part of their diagnostic activities and annually transmit results for selected bacterial species isolated from invasive infections. Participation is voluntary but supported by regional surveillance networks, ensuring progressively wider geographical representativeness."

Study size	10	Explain how the study size was arrived at	Not applicable	

Continued on next page

Quantitative variables	11	Explain how quantitative variables were handled in the analyses. If applicable, describe which groupings were chosen and why	12	"The distribution of variables was assessed using the Shapiro–Wilk test for normality of the residuals. Visual inspection was also performed using Q–Q plots and histograms with overlaid normal curves to evaluate the assumption of normality and identify potential deviations from a normal distribution."
Statistical methods	12	(*a*) Describe all statistical methods, including those used to control for confounding	11, 12	"Descriptive analyses of AMR trends were performed at both national and regional levels, evaluating percentage changes over the study period."<br>"Trend analyses were performed using separate linear regressions of resistance rates on calendar year for each Italian region"
		(*b*) Describe any methods used to examine subgroups and interactions	Not applicable	
		(*c*) Explain how missing data were addressed	12	"For regions where data are available starting from years after 2015, variations were calculated starting from the first available year, as specified. When the first available year showed no resistant pathogens isolated, the variation for that region–pathogen was not calculable."
		(*d*) *Cohort study*—If applicable, explain how loss to follow-up was addressed<br>*Case-control study*—If applicable, explain how matching of cases and controls was addressed<br>*Cross-sectional study*—If applicable, describe analytical methods taking account of sampling strategy	Not applicable	
		(*e*) Describe any sensitivity analyses	Not applicable	
**Results**				
Participants	13*	(a) Report numbers of individuals at each stage of study—eg numbers potentially eligible, examined for eligibility, confirmed eligible, included in the study, completing follow-up, and analysed	Not applicable	

		(b) Give reasons for non-participation at each stage	Not applicable	
		(c) Consider use of a flow diagram	Not applicable	
Descriptive data	14*	(a) Give characteristics of study participants (eg demographic, clinical, social) and information on exposures and potential confounders	3, 4	Results<br>Descriptive results of AMR rates in Italy
		(b) Indicate number of participants with missing data for each variable of interest	Not applicable	
		(c) *Cohort study*—Summarise follow-up time (eg, average and total amount)	Not applicable	
Outcome data	15*	*Cohort study*—Report numbers of outcome events or summary measures over time		
		Case-control study—Report numbers in each exposure category, or summary measures of exposure		
		Cross-sectional study—Report numbers of outcome events or summary measures	Not applicable	
Main results	16	(*a*) Give unadjusted estimates and, if applicable, confounder-adjusted estimates and their precision (eg, 95% confidence interval). Make clear which confounders were adjusted for and why they were included	4, 5, 6, 7	"Significant decreases were observed for MRSA ($\beta = -0.91$, 95% CI: -1.35; -0.48, $p = 0.0013$), 3GCREC ($\beta = -0.53$, 95% CI: -0.99; -0.06, $p = 0.0304$), CRKP ($\beta = -0.94$, 95% CI: -1.35; -0.53, $p = 0.0008$) and CRPA ($\beta = -0.86$, 95% CI: -1.44; -0.27, $p = 0.0095$). CRAS showed a positive but not statistically significant increase ($\beta = +0.14$, 95% CI: -1.11; 1.40, $p = 0.7977$). Conversely, VRE-faecium showed a statistically significant annual increase ($\beta = +2.76$, 95% CI: 2.58; 2.94, $p < 0.0001$)."<br>See also paragraph "Trend of AMR rates from 2015 to 2024, by region"
		(*b*) Report category boundaries when continuous variables were categorized	Not applicable	
		(*c*) If relevant, consider translating estimates of relative risk into absolute risk for a meaningful time period	Not applicable	

Continued on next page

Other analyses	17	Report other analyses done—eg analyses of subgroups and interactions, and sensitivity analyses	Not applicable	
**Discussion**				
Key results	18	Summarise key results with reference to study objectives	8	"AMR trends in Italy emerging from our analysis reveal a complex epidemiological landscape characterized by both encouraging progress and persistent challenges. Overall, resistance rates declined over the study period for five of the six priority pathogens (CRKP, 3GCREC, CRPA, MRSA, and CRAS), although CRAS resistance remained alarmingly high (always ≥74%). In contrast, VRE-*faecium* exhibited a continuous increase, representing the most concerning finding of this analysis. Comparison between 2023 and 2024 also showed a decrease for all but 3GCREC and VRE-*faecium* that increased.<br>Despite these improvements, resistance rates remain substantially high."<br>"Regional differences highlight uneven AMR distribution nationwide, with north-ern regions showing greater adherence to antimicrobial stewardship and infection prevention, resulting in better outcomes and lower antibiotic use [27]. Even though 2024 data show some exceptions in northern regions (e.g., VRE-*faecium* in Piemonte (36.0%) and in Valle d'Aosta (42.1%); MRSA (27.8%) in Lombardia), southern and centre regions generally exhibited higher resistance rates for most pathogens, both in long-term trends and in the most recent years."
Limitations	19	Discuss limitations of the study, taking into account sources of potential bias or imprecision. Discuss both direction and magnitude of any potential bias	9, 10	"This study should be interpreted considering certain limitations. First, regional participation in the AR-ISS surveillance remains heterogeneous, potentially affecting national representativeness and bias estimates toward regions with more robust surveillance capacity. Differences in AMR awareness may influence surveillance intensity and diagnostic practices, as well as testing practices, with some microorganisms being tested less frequently than others, and testing

				may be preferentially performed in clinical contexts associated with a higher suspicion of AMR. This appears to be the case for CRAS. Moreover, differences in laboratory methodologies, such as testing panels, may also introduce variability. Second, substantial regional variations in data availability, particularly in earlier years of detection, limit the precision of longitudinal trend analyses and may reduce the ability to fully capture non-linear changes over time. Our study period also encompasses the COVID-19 pandemic, which likely influenced both antimicrobial resistance patterns and surveillance activities. Third, the absence of patient-level clinical outcomes prevents direct assessment of the impact of AMR trends on individual outcomes, limiting causal interpretation.”
Interpretation	20	Give a cautious overall interpretation of results considering objectives, limitations, multiplicity of analyses, results from similar studies, and other relevant evidence	8, 9	“Similarly, the average European prevalence of VRE-*faecium* was 16.5%, compared with 34.9% in Italy [21].”<br>“Differences in laboratory participation, surveillance organization, laboratory capacity, and reporting practices may therefore contribute to the observed heterogeneity, alongside genuine epidemiological differences between regions [28].**”**<br>“Even though modest decline has been observed, the recent increase in 3GCREC and VRE-faecium may signal a reversal of previous gains, also reflecting the residual impact of the COVID-19 pandemic on surveillance and stewardship activities [30,31].”
Generalisability	21	Discuss the generalisability (external validity) of the study results	10	“Finally, this study focuses on the Italian context and its regional organization, and it is not directly generable to other EU countries and settings. However, it is important to recognize that Italy is the third most populous country in EU, and its territory encompass large and highly heterogeneous populations. Some Italian regions (e.g., Lombardia) are comparable to entire EU countries (e.g. Belgium or Portugal) in terms of population size and economic output, underscoring that they represent territories with substantial

				demographic and organizational complexity, which need to be analysed and addressed individually.”
**Other information**				
Funding	22	Give the source of funding and the role of the funders for the present study and, if applicable, for the original study on which the present article is based	12	“Funding: The authors declare they have not received funding to conduct this study.”

*Give information separately for cases and controls in case-control studies and, if applicable, for exposed and unexposed groups in cohort and cross-sectional studies.

Note: An Explanation and Elaboration article discusses each checklist item and gives methodological background and published examples of transparent reporting. The STROBE checklist is best used in conjunction with this article (freely available on the Web sites of PLoS Medicine at http://www.plosmedicine.org/, Annals of Internal Medicine at http://www.annals.org/, and Epidemiology at http://www.epidem.com/). Information on the STROBE Initiative is available at www.strobe-statement.org.
